# A Glycopeptide Dendrimer Inhibitor of the Galactose-Specific Lectin LecA and of *Pseudomonas aeruginosa* Biofilms**

**DOI:** 10.1002/anie.201104342

**Published:** 2011-09-14

**Authors:** Rameshwar U Kadam, Myriam Bergmann, Matthew Hurley, Divita Garg, Martina Cacciarini, Magdalena A Swiderska, Cristina Nativi, Michael Sattler, Alan R Smyth, Paul Williams, Miguel Cámara, Achim Stocker, Tamis Darbre, Jean-Louis Reymond

**Affiliations:** Department of Chemistry and Biochemistry, University of BerneFreiestrasse 3, 3012 Berne (Switzerland); School of Molecular Medical Sciences, University of NottinghamNottingham NG7 2UH (UK); School of Clinical Sciences, University of NottinghamNottingham NG7 2UH (UK); Institute of Structural Biology, Helmholtz Zentrum München and Center for Integrated Protein Science Munich, Departement Chemie, Technische Universität MünchenLichtenbergstrasse 4, 85747 Garching (Germany); Dipartimento di Chimica, Polo Scientifico e Tecnologico, Universita' degli Studi di FirenzeVia della Lastruccia 3, 13, 50019 Sesto Fiorentino—Firenze (Italy)

**Keywords:** biofilms, dendrimers, glycopeptides, lectins, multivalency

The spread of antibiotic resistant bacteria is one of the most pressing problems in human health today.[1] In the case of the opportunistic pathogen *Pseudomonas aeruginosa*, which causes lethal airway infections in cystic fibrosis and immunocompromised patients, the formation of biofilms plays an important role in antibiotic resistance and disease progression.[2] Biofilm formation is mediated in part by the galactose-specific lectin LecA (PA-IL)[3] and the fucose-specific lectin LecB (PA-IIL),[4] as evidenced by studies with deletion mutants[5] and the partial inhibitory effect of simple fucose and galactose derivatives in vitro and in vivo.[5a, 6] Understanding the glycoconjugate–lectin interaction is a key feature in developing potent biofilm inhibitors. Capitalizing on the well-known cluster effect observed on binding of multivalent carbohydrates to lectins,[7–11] we recently reported the first case of *P. aeruginosa* biofilm inhibition with a multivalent lectin inhibitor, the fucosylated glycopeptide dendrimer FD2 (cFuc-Lys-Pro-Leu)_4_ (*Lys-*Phe-Lys-Ile)_2_*Lys-*His-IleNH_2_, which targets LecB.[12, 13] Herein we report the first case of *P. aeruginosa* biofilm inhibition with a multivalent ligand targeting the galactose-specific lectin LecA, using the related β-phenylgalactosyl peptide dendrimer GalAG2.

**Scheme 1:**
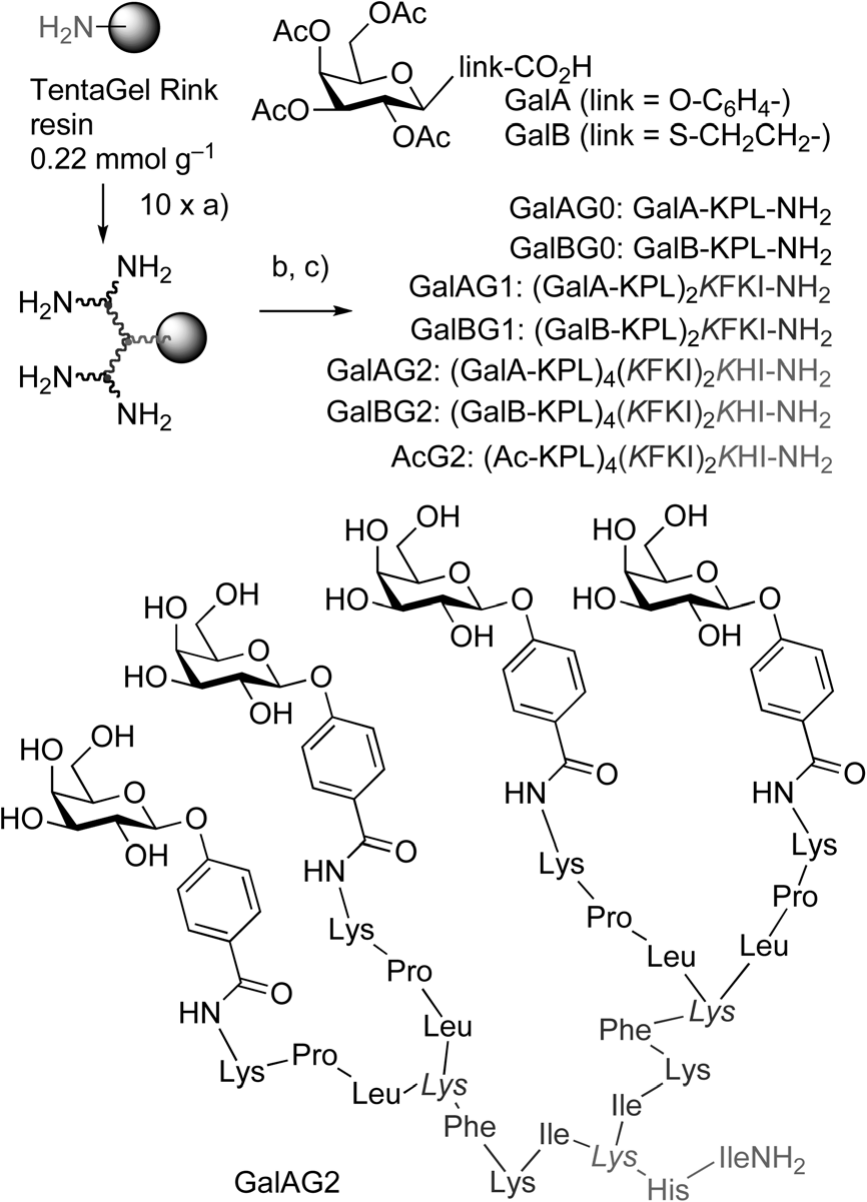
Synthesis of the galactosyl peptide dendrimers: a) FmocAAOH, PyBOP, DIEA, NMP, 1–3 h (2×), then 20 % piperidine in DMF, (2×10 min), b) GalA or GalB, HCTU, DIEA, NMP, or acetylation c) MeOH/NH_3_/H_2_O (v/v 8:1:1), then TFA/TIS/H_2_O (95:2.5:2.5). Fmoc=9-fluorenylmethyloxycarbonyl, PyBOP=1-benzotriazolyloxy-tris(pyrollidino)phosphonium, DIEA=diisopropylethylamine, NMP=*N*-methylpyrrolidone, DMF=*N,N*-dimethylformamide, HCTU=2-(6-Chloro-1-H-benzotriazole-1-yl)-1,1,3,3-tetramethylaminium hexafluorophosphate, TFA=trifluoroacetic acid, TIS=triisopropylsilane, *Lys*=lysine as branching unit, K=lysine (Lys), P=proline (Pro), L=leucine (Leu), F=phenylalanine (Phe).

Considering the favorable properties of FD2 as lectin inhibitor, we set out to investigate if its peptide dendrimer portion might also be suitable for inhibitors of the galactose-specific *P. aeruginosa* lectin LecA. Because hydrophobic groups in the sugar anomeric position have been shown to enhance the affinity of galactosides to LecA,[3b, 14] acetyl-protected 4-carboxyphenyl β-galactoside (GalA) was attached to the peptide dendrimer. To probe the effect of the sugar-dendrimer linker on binding, carboxypropyl β-thiogalactoside (GalB) was also introduced as the last building block in solid-phase peptide synthesis to provide dendrimers GalA/BG1 and GalA/BG2, and the linear peptides GalA/BG0. The dendrimers were obtained pure as trifluoroacetate salts after deacetylation on a solid support, acid-mediated cleavage from the support, and purification by preparative HPLC (Scheme 1).

The binding affinity to LecA was evaluated in a hemagglutination assay that measured the inhibition of LecA-induced agglutination of rabbit erythrocytes in comparison to d-galactose as the reference.[15] Thermodynamic parameters were obtained by isothermal titration calorimetry (ITC). A strong multivalency effect on binding was observed in both the GalA and the GalB dendrimer series. The strongest effect occurred with dendrimer GalAG2, which showed a 4000-fold increase in hemagglutination inhibition activity and a 875-fold increase in binding (*K*_d_) to LecA compared to d-galactose (Table 1). Considering that the number of sugars and the peptidic scaffold are the same in both series, the presence of the phenyl group in GalA dendrimers led to a remarkable enhancement in affinity compared with the thiogalactoside GalB dendrimers.

**Table 1:** Data for binding to *P. aeruginosa* lectin LecA

		Hemagglutination assay[a]		Isothermal titration calorimetry (ITC)[b]					
Ligand	*n*	MIC [μm]	r.p./*n*	*n*′	Δ*H* [kcal mol^−1^]	−*T* Δ*S* [kcal mol^−1^]	Δ*G* [kcal mol^−1^]	*K*_d_ [μm]	r.p./*n*
d-galactose	1	3125	1	1.1±0.1	−8.4±0.1	2.3±0.4	−6.0±0.3	87.5±3.5	1
*p*-nitrophenyl-β-galactoside (NPG)	1	550	5.7	0.9±0	−10.0±0.1	3.4±0.1	−6.6±0	14.1±0.2	6.2
isopropyl-β-thiogalactoside (IPTG)	1	1100	2.8	1.1±0.1	−8.9±0.5	2.8±0.5	−6.1±0.1	32.4±2.7	2.7
GalAG0 (GalA-KPL-NH_2_)	1	80	40	1.0±0.1	−10.8±0.6	3.4±0.7	−7.4±0.1	4.2±0.9	20.9
GalAG1 (GalA-KPL)_2_*K*FKI-NH_2_	2	31	50	2.6±0.3	−11.5±0.7	2.9±0.9	−8.7±0.2	0.5±0.2	91.1
GalAG2 (GalA-KPL)_4_(*K*FKI)_2_*K*HI-NH_2_	4	0.78	1000	4.2±0.6	−12.0±1.4	2.6±1.5	−9.4±0	0.1±0.01	219
GalBG0 (GalB-KPL-NH_2_)	1	2500	1.3	1.2±0.1	−7.3±1.0	1.5±1.1	−5.9±0.1	51.5±6.7	1.7
GalBG1 (GalB-KPL)_2_*K*FKI-NH_2_	2	630	2.5	2.5±0.3	−8.3±0.4	0.7±0.4	−7.6±0.1	2.1±1.0	20.5
GalBG2 (GalB-KPL)_4_(*K*FKI)_2_*K*HI-NH_2_	4	125	12.5	4.3±0.2	−9.1±0.2	0.3±0.0	−8.8±0.2	0.4±0.1	59.9

[a] MIC=minimal inhibitory concentration for the hemagglutination assay. Conditions: twofold serial dilutions of the tested compounds were incubated with the LecA lectin for 30 min at 4 °C, after which time rabbit erythrocytes (5 % solution in PBS) were added and further incubated for another hour at RT. The MIC corresponds to the highest dilution causing a complete inhibition of hemagglutination. *n*=number of galactose residues per ligand, r.p./*n*=relative potency per galactose residue=(MIC_(D-galactose)_/MIC_(ligand)_)/*n*.

[b] Stoichiometry *n*′=number of occupied lectin galactose binding sites per dendrimer; thermodynamic parameters and dissociation constant *K*_d_ reported as an average of two independent runs from ITC in 0.1 m tris(hydroxymethyl)aminomethane (Tris base), pH 7.5, 25 mm CaCl_2_, 25 °C, r.p./*n*=(*K*_*d*(d-galactose)_/*K*_*d*(ligand)._)/*n*. The acetylated G2 dendrimer AcG2 did not show any measurable affinity in ITC (Figure S26 in the Supporting Information).

**Figure 1:**
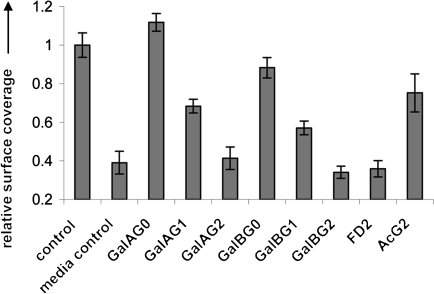
Inhibition of *P. aeruginosa* wild-type strain PAO1 biofilms by glycopeptide dendrimers. Biofilms were grown on steel coupons inoculated with PAO1 for 48 h at 37 °C in the presence of ligands (20 μm galactosyl endgroup) followed by staining with acridine orange prior to analysis of surface coverage. Metal coupons incubated with bacteria only (control), growth media with no bacteria (media control), and with FD2 dendrimer, were used as positive and negative controls.

The dendrimers inhibited *P. aeruginosa* biofilms, as evidenced by using the steel coupon assay (Figure 1).[16] A generation-dependent effect was observed in both the GalA and the GalB series, with essentially complete inhibition of biofilm formation with the second-generation (G2) dendrimers, as observed with the LecB inhibitor FD2, whereas the acetylated dendrimer AcG2, which lacks galactosyl groups, showed only a small inhibition of biofilm formation. Bacterial growth was unaffected by the ligands, thus ruling out toxicity effects. These data were consistent with a LecA-mediated inhibition of *P.* *aeruginosa* biofilms by the dendrimers.

The origin of the binding affinity of the glycopeptide dendrimers for LecA was investigated further. Multivalency effects provided for increased potency compared to the monovalent ligands in both the GalA and the GalB series, as indicated by the ratio *K*_*d*GalAG0_/*K*_*d*GalAG2_=42 and *K*_*d*GalBG0_/*K*_*d*GalBG2_=129. This effect was of the same order of magnitude as that previously observed with FD2 and LecB (FD2/G0=40),[13c] and probably reflects a favorable presentation of the glycosides by the peptide dendrimers for lectin binding. The presence of the aromatic β-phenyl aglycone in the GalA series caused an additional affinity increase, as indicated by the ratios *K*_*d*GalBG0_/*K*_*d*GalAG0_=12, *K*_*d*GalBG1_/*K*_*d*GalAG1_=4, *K*_*d*GalBG2_/*K*_*d*GalAG2_=4 (MIC values: MIC_GalBG0_/MIC_GalAG0_=32, MIC_GalBG1_/MIC_GalAG1_=21, MIC_GalBG2_/MIC_GalAG2_=160). As normally observed for lectin–carbohydrate interactions,[17] LecA–galactoside binding was enthalpically driven, with slightly unfavorable entropic contributions (Table 1).

The structures of the lectin–ligand complexes were investigated to gain insight into the molecular basis of glycopeptide dendrimer–lectin interactions. While heavy precipitates formed in all crystallization trials using the G1 and G2 dendrimers, good quality crystals were obtained for complexes of LecA with the monovalent ligands NPG, GalAG0, and GalBG0, and their X-ray structures were determined.

**Figure 2:**
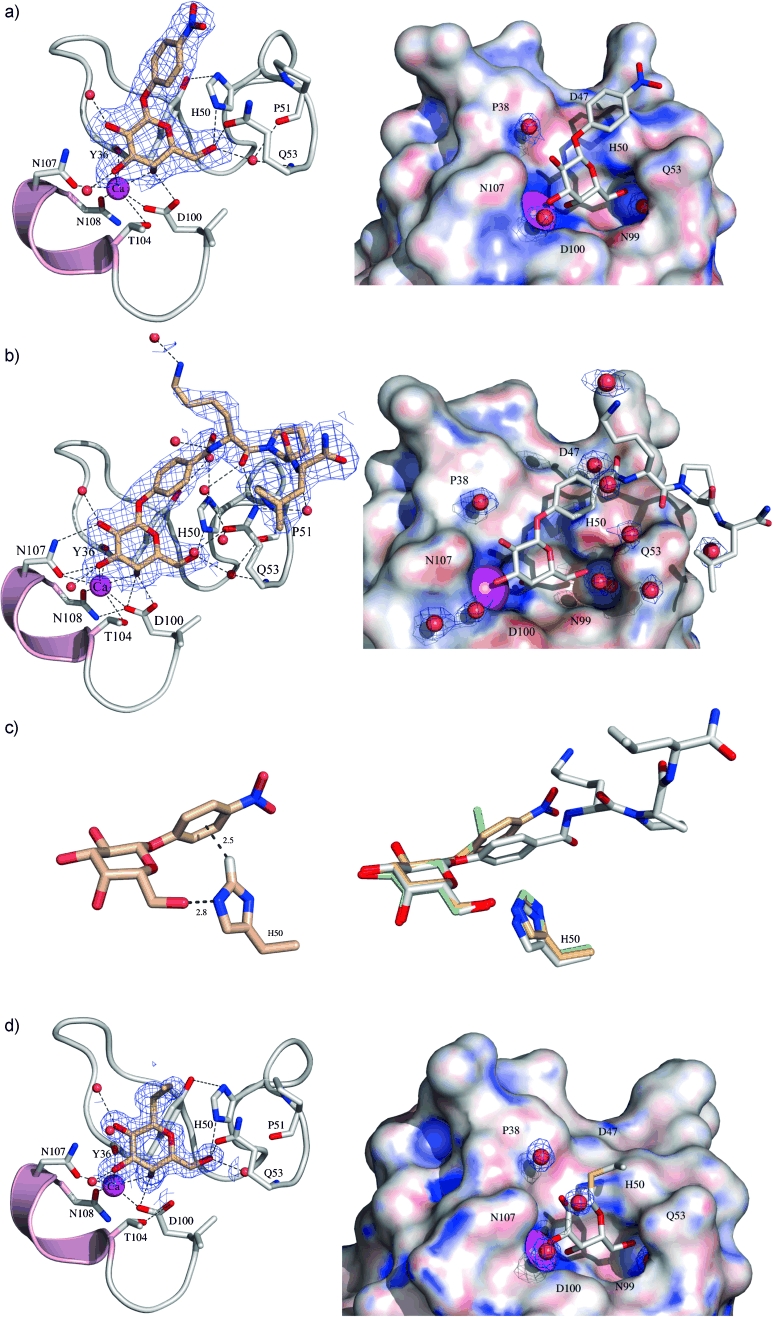
Structures of cocrystallized ligands (in sticks) a) NPG, b) GalAG0, and d) GalBG0 with LecA. The fit of the ligands to the electron density map is shown in the left-hand panel. Noncovalent interactions between the ligand and the protein are shown by dotted lines. The well-defined electron density for water molecules (red spheres) is shown in the right-hand panel. The protein is shown as a surface model colored according to electrostatic potentials ranging from −2 kcal mol^−1^ (red) to +2 kcal mol^−1^ (blue). c) Left: T-shaped interactions of the imidazole side chain of His50 of LecA with NPG. Right: Overlay of LecA in complex with NPG (brown), GalAG0 (gray), and GalBG0 (green). D=aspartic acid (Asp), H=histidine (His), N=asparagine (Asn), Q=glutamine (Gln), Y=tyrosine (Tyr), T=threonine (Thr). Atom labels: N blue, O red.

In the structure of the NPG–LecA complex, the galactosyl group binds in the same orientation as free galactose, with the characteristic coordination of the C(3)–OH and C(4)–OH groups to the lectin-bound calcium ion (Figure 2 a).[18a] The nitrophenyl group occupies a hydrophobic groove adjacent to the glycoside binding site, with van der Waals contacts to residues Tyr36, Pro38, and His50. Residue His50 not only forms a hydrogen bond with the C(6)–OH group of the galactose, as observed in other LecA-galactose complexes,[18] but also engages in a T-stack interaction with the nitrophenyl group. The C(ɛ)–H group of the imidazole ring is located 2.5 Å from the phenyl ring and points towards it. Similarly, the structure of the GalAG0–LecA complex shows the phenyl group in the hydrophobic groove adjacent to the carbohydrate binding site and the “face-to-edge” interaction with the C(ɛ)–H group of His50 (Figure 2 b).

Binding of the tripeptide portion of GalAG0 to LecA causes an average 0.66 Å inward movement of the phenyl linker relative to the position of the nitrophenyl moiety of NPG. This movement triggers concomitant inward movements of the interacting Glu49-His50-Pro51 loop of 0.45 Å on LecA. Overall, tighter surface interactions between LecA and GalAG0 are observed relative to NPG (Figure 2 c). Analysis of aromatic His⋅⋅⋅X interactions by examining 593 PDB structures indicates that “face-to-edge” stack interactions are frequent arrangements for His⋅⋅⋅X interacting pairs, with the X partners representing aromatic side chains of the same protein.[19] However, an intermolecular T-stack of an aromatic ring with the C(ɛ)–H group of the histidine imidazole side chain at a distance of 2.5 Å is unprecedented. This interaction probably contributes favorably to the binding of the phenyl galactoside to the lectin.

In the structure of the GalBG0–LecA complex, solely the galactosyl group and the thioethyl part of its linker are well-resolved (Figure 2 d). Interestingly, the linker moiety points perpendicularly away from the protein surface in GalBG0, thus indicating impaired surface contacts of its tripeptide moiety with LecA.

**Figure 3:**
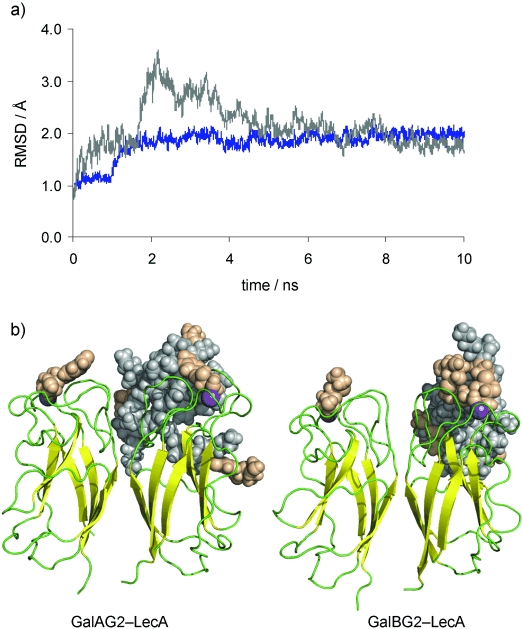
a) Plot of root-mean-square deviation (RMSD) against time for the MD simulations of GalAG2–LecA (blue) and GalBG2–LecA (gray) complexes. b) Final models of GalAG2–LecA and GalBG2–LecA complexes obtained from the last snapshots of MD simulations. The protein is shown in cartoon representation and the glycopeptide dendrimers as CPK models (beige: phenyl/thio galactoside moieties, gray: rest of the dendrimer). The Ca^2+^ ions are shown in magenta and indicate the location of the galactose binding pocket.

A molecular dynamics (MD) study was carried out to gain insight into the possible cause of the multivalency effects that might explain the stronger binding of GalA/BG2 to LecA compared to their monovalent ligands. Models of GalA/BG2 bound to the lectin were constructed by fusing the MD-simulated dendrimer structures with the experimentally determined structures of the GalAG0–LecA and GalBG0–LecA complexes, followed by energy minimization and simulations for a period of 10 ns (Figure 3 a). In the resulting complexes, the dendrimers appeared too small to allow bridging of two galactose binding sites within the same LecA dimer. However, the galactoside residues were exposed on the surface (Figure 3 b). This observation suggests that the enhanced binding by multivalency could result from the ability of the dendrimers to bridge different LecA tetramers in addition to secondary interactions within the LecA–dendrimer complex.

In summary, we have reported the first example of *P. aeruginosa* biofilm inhibition with multivalent galactosylated LecA ligands.[20] The strongest binding was observed with the second-generation glycopeptide dendrimer GalAG2. This dendrimer contains an aromatic aglycone linker that engages in an unprecedented T-stack interaction with His50 at the LecA galactose binding site, as evidenced by X-ray crystallography. This interaction enables additional contacts between the outer tripeptide branch of the dendrimer and the lectin; the contacts do not occur in the case of the thiopropyl linker in the GalB-type ligands, for which the tripeptide is disordered in the X-ray structure. Interestingly, both GalAG2 and GalBG2 dendrimers displayed potent biofilm inhibition, whereas the G1 analogues were much less active and the G0 analogs were inactive. Thus multivalency played a much more important role for biofilm inhibition than the nature of the linker. Future experiments will address activity improvement by dendrimer sequence optimization and the synthesis of analogues with higher multivalency.
