# Supplementary material for: A Glycopeptide Dendrimer Inhibitor of the Galactose-Specific Lectin LecA and of Pseudomonas aeruginosa Biofilms
Source: Angew Chem Int Ed Engl. 2011 Sep 14;50(45):10631–5. doi: 10.1002/anie.201104342 (PMC3262149; doi:10.1002/anie.201104342)
Supplement: Supplementary file 1 [file anie0050-10631-SD1.pdf]

Angewandte Chemie
Eine Zeitschrift der Gesellschaft Deutscher Chemiker

Supporting Information
© Wiley-VCH 2011
69451 Weinheim, Germany

A Glycopeptide Dendrimer Inhibitor of the Galactose-Specific Lectin LecA and of *Pseudomonas aeruginosa* Biofilms**

*Rameshwar U. Kadam, Myriam Bergmann, Matthew Hurley, Divita Garg, Martina Cacciarini, Magdalena A. Swiderska, Cristina Nativi, Michael Sattler, Alan R. Smyth, Paul Williams, Miguel Cámara, Achim Stocker, Tamis Darbre, and Jean-Louis Reymond**

anie_201104342_sm_miscellaneous_information.pdf

Supporting Information for:

A Glycopeptide dendrimer inhibitor of the Galactose Specific Lectin LecA and of *Pseudomonas aeruginosa* Biofilms**

Rameshwar U. Kadam,[a)] Myriam Bergmann,[a)] Matthew Hurley,[b)c)] Divita Garg,[d)] Martina Cacciarini,[e)] Magdalena A.Swiderska,[a)] Cristina Nativi,[e)] Michael Sattler,[d)] Alan R. Smyth,[c)] Paul Williams,[b)] Miguel Cámara,[b)] Achim Stocker,[a)] Tamis Darbre [a)] and Jean-Louis Reymond [a)] *

[a]Department of Chemistry and Biochemistry, University of Berne, Freiestrasse 3, CH-3012, Berne, Switzerland, Fax: (+41) 31 631 80 57; E-mail: jean-louis.reymond@ioc.unibe.ch
[b]School of Molecular Medical Sciences, University of Nottingham, Nottingham NG7 2RD, UK
[c]School of Clinical Sciences, University of Nottingham NG7 2UH, U.K
[d]Institute of Structural Biology, Helmholtz Zentrum München and Center for integrated Protein Science Munich at Dept Chemie, Technische Universität München, Lichtenbergstr. 4, 85747 Garching, Germany
[e]Dipartimento di Chimica, Polo Scientifico e Tecnologico, Universita' degli Studi di Firenze, Via della Lastruccia 3, 13, I-50019 Sesto Fiorentino – Firenze, Italy

Table of Contents

CARBOHYDRATE DERIVATIVES SYNTHESIS 2
DENDRIMER SYNTHESIS 6
BIOFILM ASSAYS 14
P. AERUGINOSA LECTIN LECA EXPRESSION AND PURIFICATION 15
HEMAGGLUTINATION ASSAY 17
ISOTHERMAL TITRATION CALORIMETRY (ITC) 19
X-RAY CRYSTALLOGRAPHY 22
MOLECULAR DYNAMIC SIMULATIONS 28
REFERENCES 32

Carbohydrate Derivatives Synthesis

3-(Tetra-O-acetyl-β-D-galactopyranosylthio)-propionic acid (GalB). To a solution of β-D-galactose pentaacetate (3 g, 7.69 mmol) in dry CH_2Cl_2 (45 mL) was added 3-mercaptopropionic acid (2.68 mL, 30.74 mmol, 4 eq.) and $BF_3.Et_2O$ (1.42 mL, 11.53 mmol, 1.5 eq.). The solution was stirred for 90 min. at r.t. Washing vigorously with cold water and brine, afforded compound **GalB** (2.67 g, 6.13 mmol, 79%) as a colorless oil. ^{1}H NMR ($CDCl_3$, 300 MHz): δ= 1.90 (s, 3H), 2.68 (s, 3H), 2.70 (s, 3H), 2.14 (s, 3H), 2.73 (t, J= 7.35 Hz, 2H), 2.83-3.01 (m, 2H), 3.88 (t, J= 6.6 Hz, 1H), 4.03-4.17 (m, 2H), 4.50 (d, J= 9.96 Hz, 1H), 5.00 (dd, J= 3.39 and 9.96 Hz, 1H), 5.16 (t, J= 9.78 Hz, 1H), 5.39 (d, J= 2.82 Hz, 1H). ^{13}C NMR ($CDCl_3$, 300 MHz): 177.1, 170.7, 170.3, 170.2, 169.7, 84.6, 74.5, 71.9, 67.4, 67.1, 61.6, 38.3, 35.4, 25.4, 20.8, 20.7, 20.6, 19.5. EI-MS: m/z= 459 $[M+Na]^+$.

4-(Tetra-O-acetyl-β-D-galactopyranosyloxy) benzoic Acid (GalA).

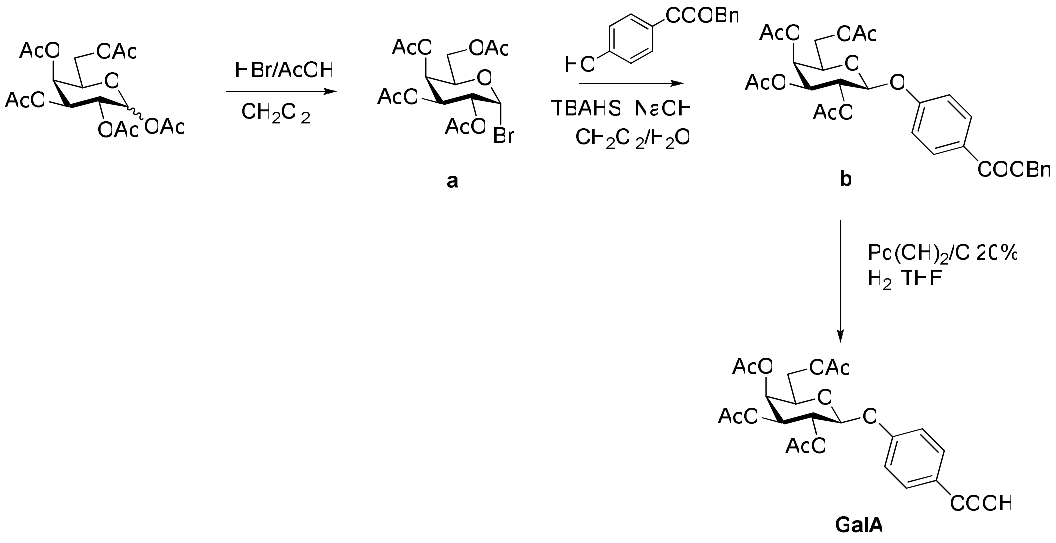

Figure S1. Synthesis of GalA.

To a solution of galactose pentaacetate (2.0 g, 5.12 mmol) in dry CH_2Cl_2 (10 mL) at 0 °C, 33% HBr/AcOH (4.5 mL) was slowly added. The resulting orange solution was stirred at r.t. for 30 min then diluted with CH_2Cl_2 (50 mL), washed with saturated aqueous $NaHCO_3$ (3 x 30 mL) and dried over Na_2SO_4. The solvent was removed under reduced pressure to give the crude α-bromo-derivative **a** (2.0 g, 5.02 mmol) which was used without purification for the glycosidation. ^{1}H-NMR (200 MHz, $CDCl_3$): δ = 6.70 (d, J = 3.9 Hz, 1H, H-1), 5.52 (dd, J = 3.2 Hz, 1.2 Hz, 1H, H-4), 5.41 (dd, J = 10.6 Hz, 3.2 Hz, 1H, H-3), 5.05 (dd, J = 10.6 Hz, 3.9 Hz, 1H, H-2), 4.52-4.45 (m, 1H, H-5), 4.24-4.06 (m, 2H, H-6, H-6'), 2.15 (s, 3H, Ac), 2.12 (s, 3H, Ac), 2.06 (s, 3H, Ac), 2.01 (s, 3H, Ac).

To a solution of **a** (2.0 g, 5.02 mmol), benzyl-4-hydroxy-benzoate (2.34 g, 10.3 mmol) and tetrabutylammoniumhydrogensulfate (1.74 g, 5.12 mmol) in CH_2Cl_2 (30 mL) at 0 °C a 1 M aq. solution of NaOH (10 mL) was added. The biphasic mixture was mechanically stirred at r.t. for 36 h, then diluted with CH_2Cl_2 (30 mL), washed with NaOH 1M (2 x 30 mL) and dried over Na_2SO_4. The solvent was removed under reduced pressure to give a crude which was purified by flash column chromatography (eluent: petroleum ether/ethyl acetate 2:1 and 1:1) to afford **b** (1.83 g, 3.27 mmol, 64% in two steps) as a white foam slightly contaminated by benzyl-4-hydroxy-benzoate, which can be easily removed after hydrogenation.

A solution of **b** (1.7 g, 3.04 mmol) in THF (20 mL) was hydrogenated over 20% $Pd(OH)_2/C$ (120 mg) under H_2 at r.t. for 1 h. The reaction mixture was filtered through a Celite pad, washed with fresh THF and the solvent removed under reduced pressure. The crude was purified by FCC on silica gel (eluent: CH_2Cl_2/MeOH = 9:1) to afford **GalA** (1.0 g, 2.13 mmol 70%) as a white solid. M.p. 88-91 °C. $[\alpha]_D$ –5.96 (c 0.52, MeOH). ^{1}H-NMR (400 MHz, $CDCl_3$): δ = 8.09-8.05 (m, 2H), 7.07-7.03 (m, 2H), 5.52 (dd, J = 10.4 Hz, 7.9 Hz, 1H, H-2), 5.48 (dd, J = 3.4 Hz, 0.7 Hz, 1H, H-4), 5.16 (d, J = 7.9 Hz, 1H, H-1), 5.14 (dd, J = 10.4 Hz, 3.4 Hz, 1H, H-3), 4.25-4.10 (m, 3H, H-5, H-6, H-6'), 2.19 (s, 3H, Ac), 2.070 (s, 3H, Ac), 2.066 (s, 3H, Ac), 2.02 (s, 3H, Ac). ^{13}C-NMR (100 MHz, $CDCl_3$): δ = 170.7, 170.3, 170.2, 170.1, 169.3, 160.9, 132.3, 124.0, 116.2, 98.7, 71.3, 70.7, 68.4, 66.8, 61.4, 20.7, 20.6, 20.5. ESI-MS: m/z: 467.2 $[M-1]^-$. HRMS (ESI-MS): calc. for $[C_{21}H_{24}O_{12}+H^+]$: 469.1347; found: 469.1341.

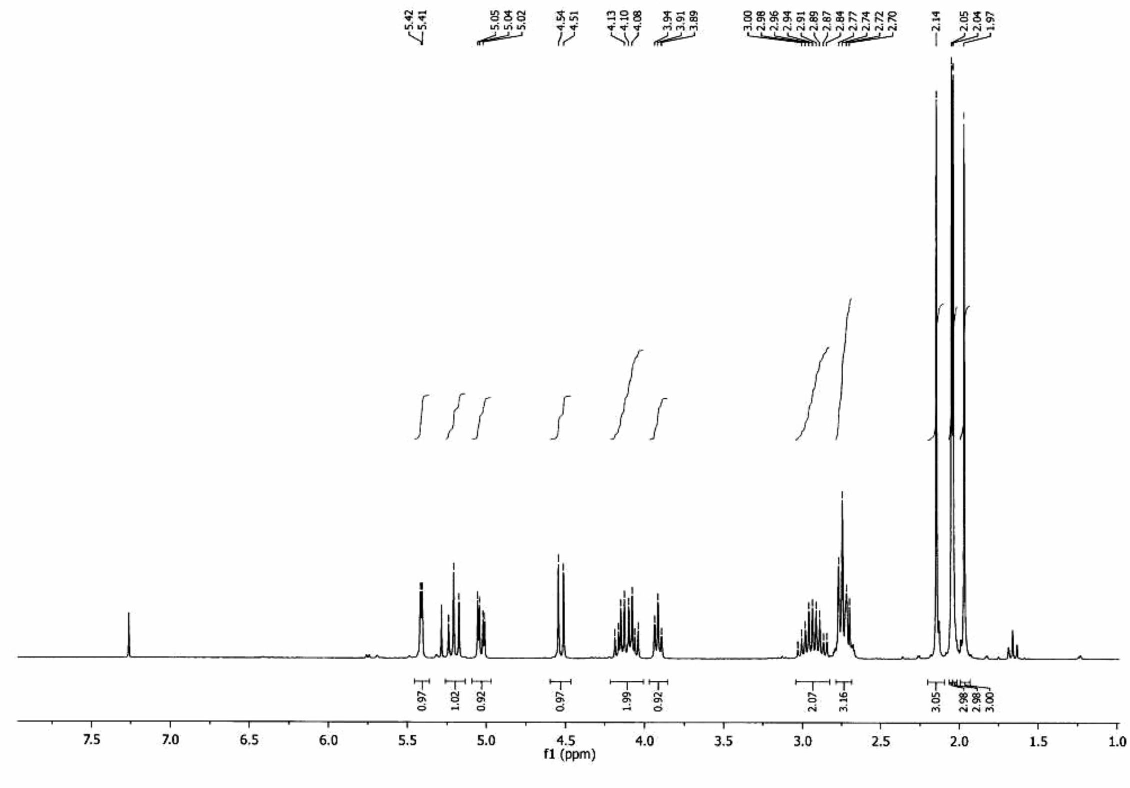

Figure S2. ^{1}H NMR of **GalB**.

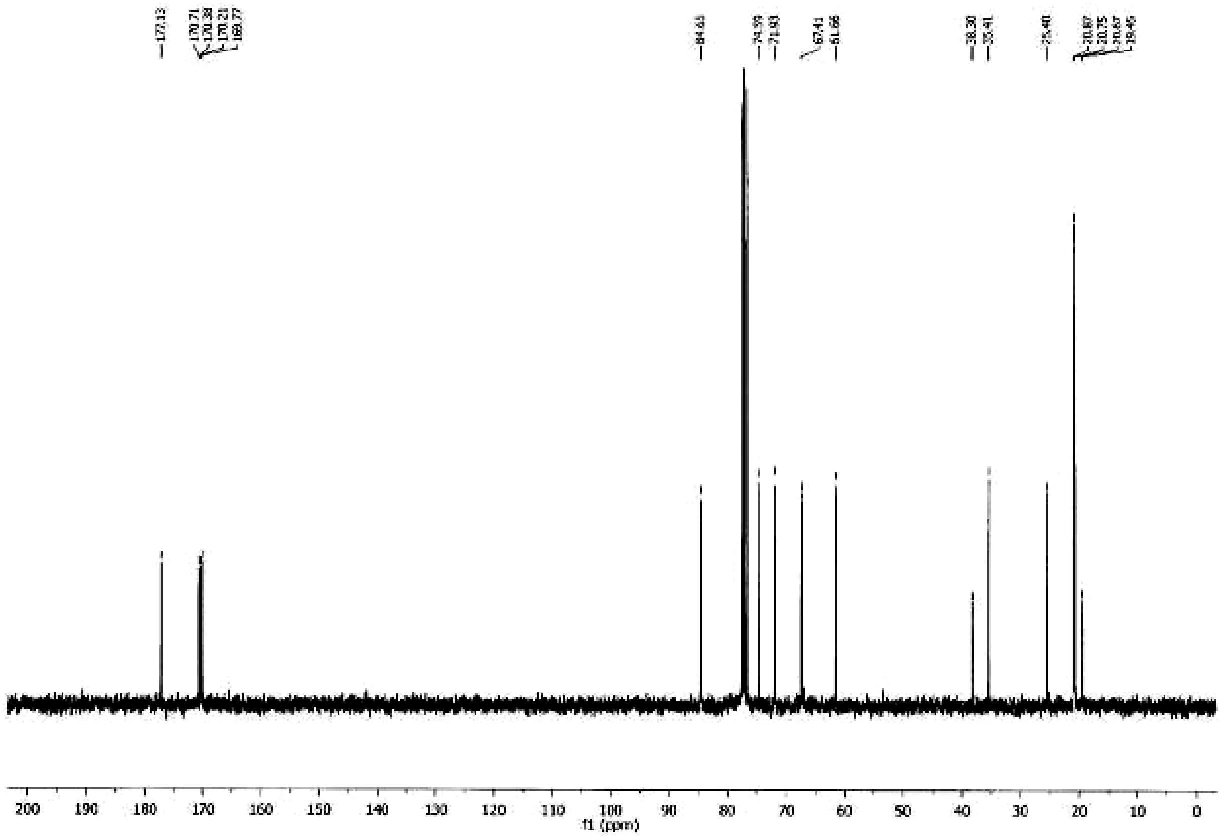

Figure S3. ^{13}C NMR of **GalB.**

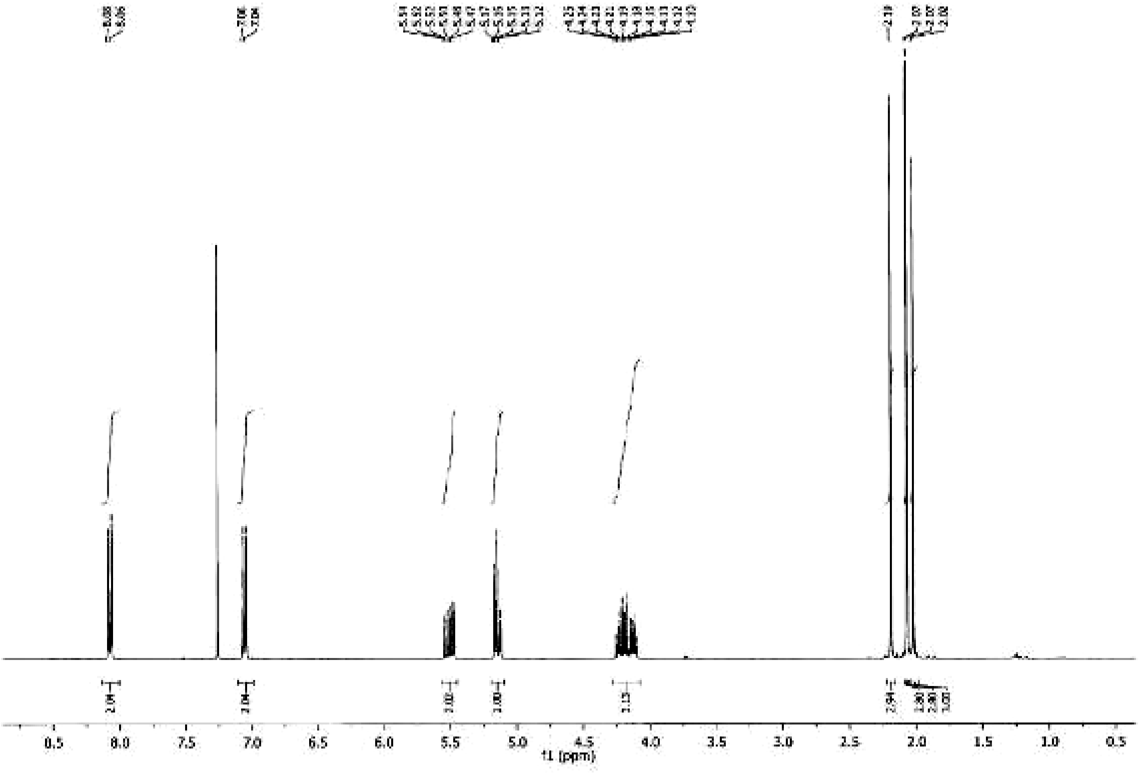

Figure S4. ^{1}H NMR of GalA.

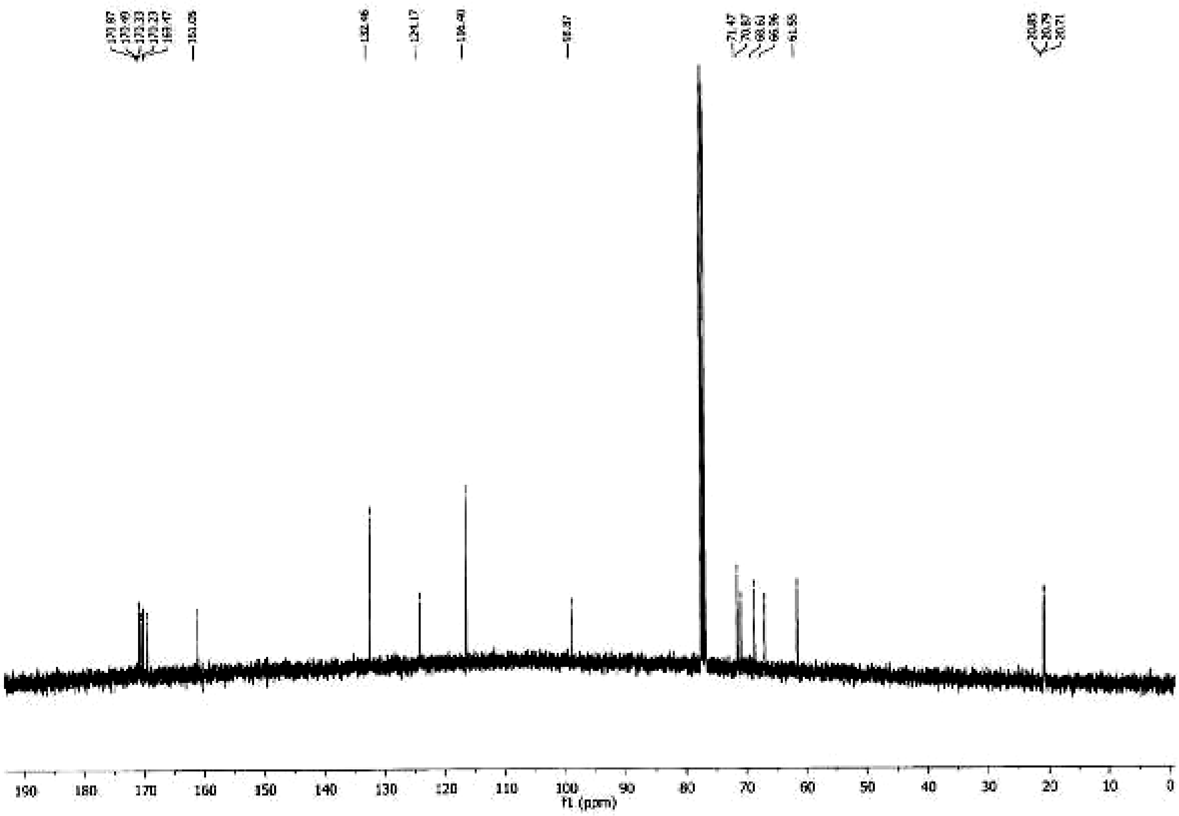

Figure S5. ^{13}C NMR of GalA.

Dendrimer Synthesis

Dendrimers and short peptides were synthesized on solid phase using Fmoc chemistry in a plastic syringes (10 mL). Rink amide NovaSyn® TGR resin (loading: 0.24 mmol/g) (purchased from Novabiochem) was acylated with each Fmoc-protected α-amino acid (3 eq) in the presence of benzotriazol-1-yl-oxytripyrrolidinophosphonium hexafluorophosphate (PyBOP) (3 eq) and *N*,*N*-Diisopropylethylamine (DIEA) (5 eq) in N-Methyl-2-pyrrolidon (NMP). The Fmoc-protected groups were removed with a solution of 20% piperidine in DMF. In the end of the sequence the terminal amino acids were coupled with the protected sugar derivative (5 eq) in the presence of DIEA (5 eq) and 2-(6-Chloro-1H-benzotriazole-1-yl)-1,1,3,3-tetramethylaminium hexafluorophosphate) (HCTU) (3 eq). The carbohydrate was deprotected with a solution of MeOH/NH_3/H_2O (v/v 8:1:1). The resin was dried and the cleavage was carried out with TFA/TIS/H2O (95:2.5:2.5). Peptides were precipitated with methyl *tert*-butyl ether and purified by preparative HPLC.

Amino acids were used as the following derivatives: Fmoc-His(Boc)-OH, Fmoc-Leu-OH, Fmoc-Phe-OH, Fmoc-Ile-OH, Fmoc-Pro-OH, Fmoc-Lys(Boc)-OH, Fmoc-Lys(Fmoc)-OH. Chemicals were used as supplied and solvents were of analytical grade. Analytical RP-UHPLC was performed in Dionex ULTIMATE 3000 RS chromatography system (ULTIMATE 3000 RS Photo diode array detector) using a Dionex Acclaim® RSLC 120 C18, 3.0 x 50 mm, particle size 2.2 µm, 120 Å pore size, flow rate 1.2 ml min^{-1} column. Compounds were detected by UV absorption at 214 nm. Preparative RP-HPLC was performed with HPLC-grade acetonitrile and MilliQ deionized water using a Dr. Maisch GmbH Reprospher C18-DE, 100 x 30 mm, particle size 5 µm, 100 Å pore size column installed on a Waters Prep LC Controller sytem (flow rate 60 ml min^{-1}, gradient 0.72 ml min^{-1} CH_3CN). Eluent A contained water and 0.1% TFA; eluant B contained acetonitrile, water, and TFA (3/2/0.1%). MS spectra were provided by the Service of Mass Spectrometry of the Department of Chemistry and Biochemistry, University of Bern.

GalAG0: GalA-Lys-Pro-LeuNH$_2$ (20.9 mg , 55 %). MS (ESI+) calc for $C_{30}H_{47}N_5O_{10}$ $[M+H]^+$: 638.33, found 638.2

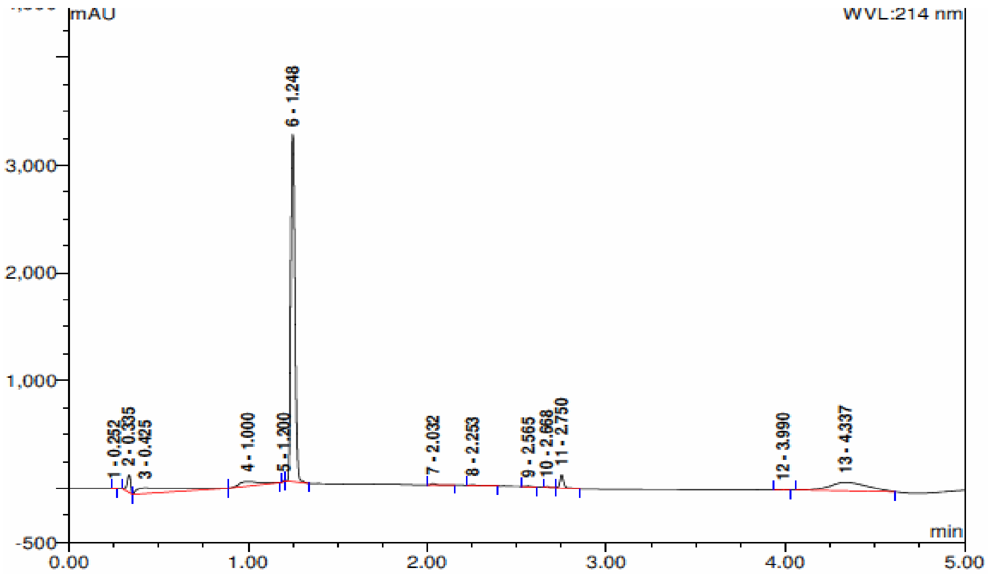

Figure S6. Analytical RP-UHPLC of **GalAG0**.

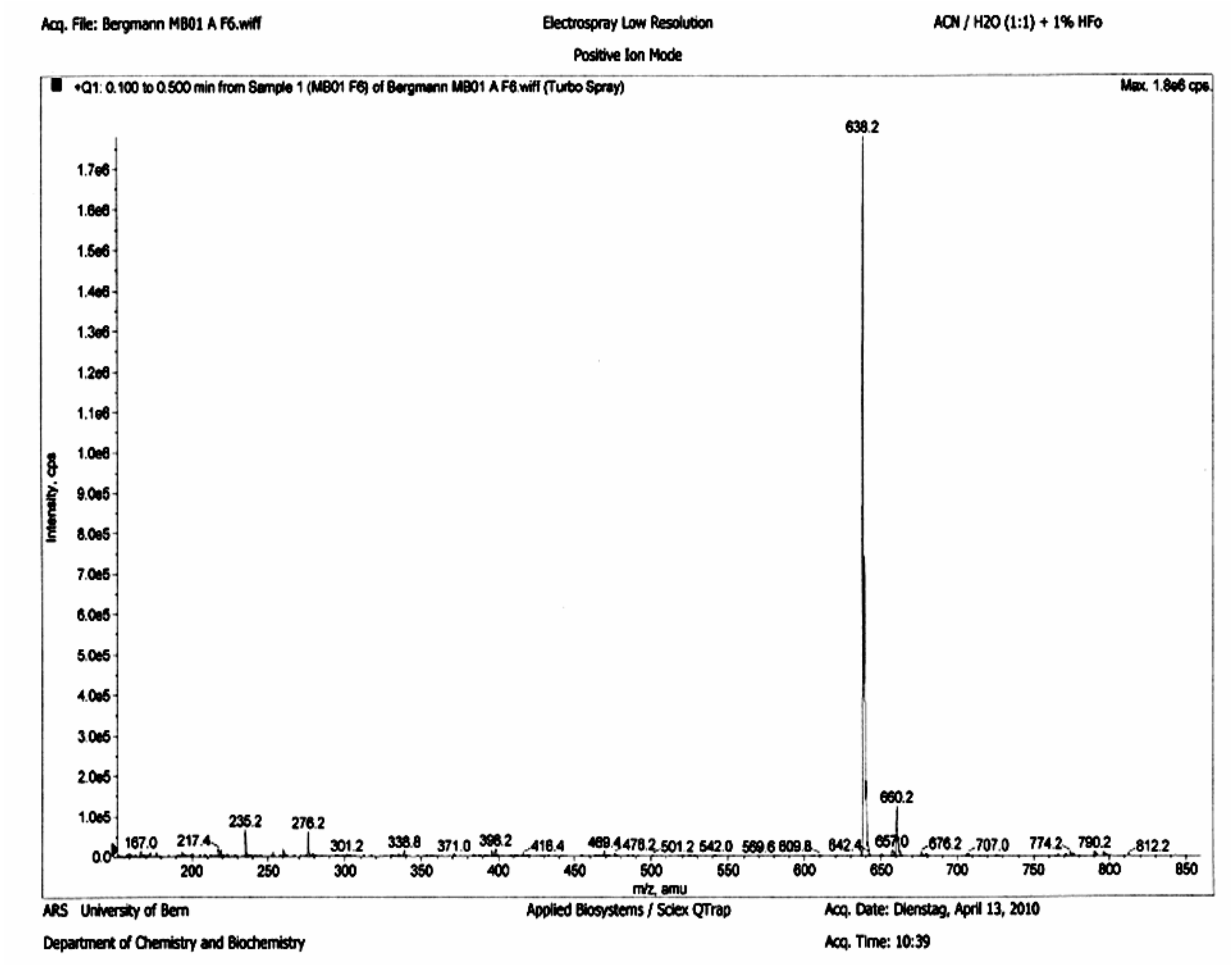

Figure S7. ESI-MS of **GalAG0.**

GalBG0: GalB-Lys-Pro-LeuNH$_2$ (11 mg, 30%). MS (ESI+) calc for $C_{26}H_{47}N_5O_9S$ [M+H]+: 606.31, found 606.2.

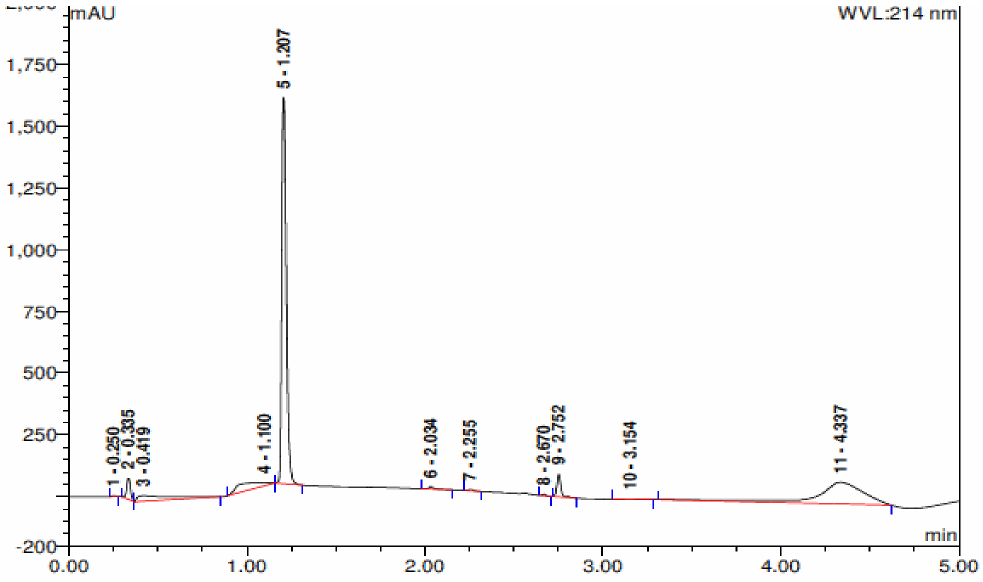

Figure S8. Analytical RP-UHPLC of **GalBG0.**

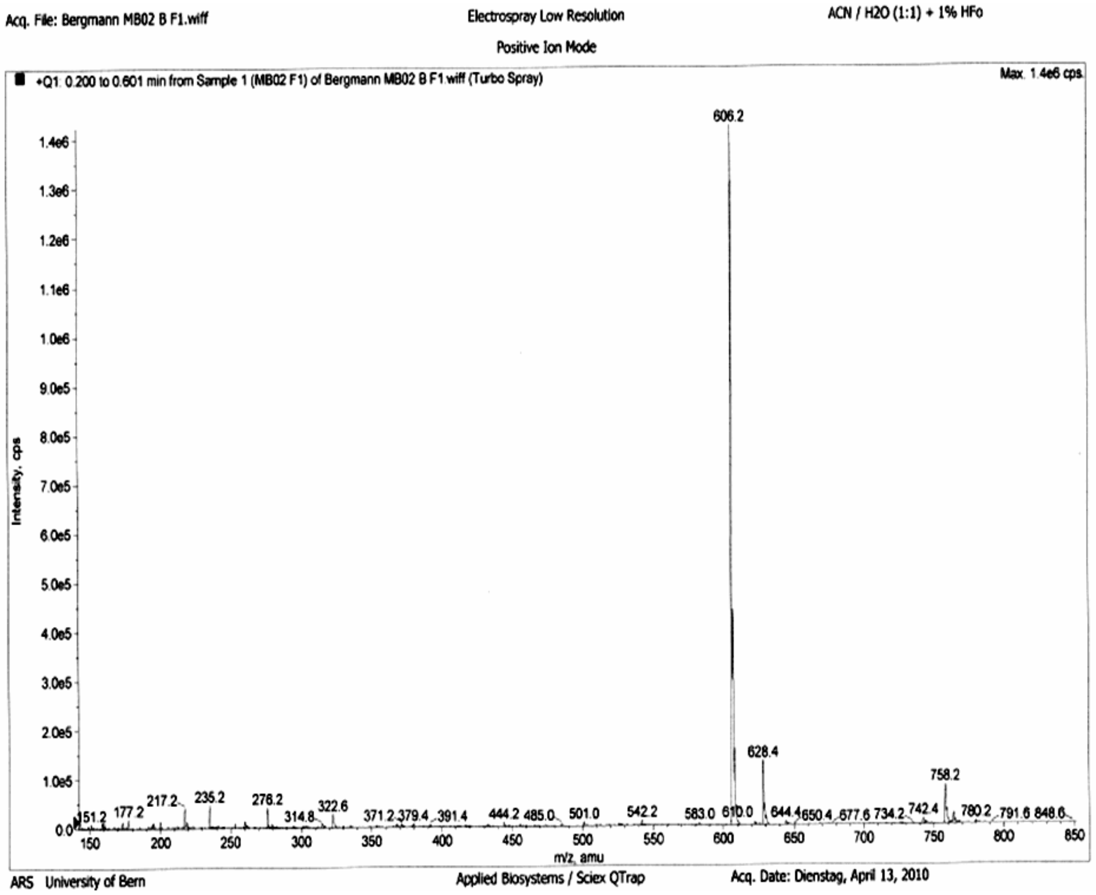

Figure S9. ESI-MS of **GalBG0.**

GalAG1: (GalA-Lys-Pro-Leu)$_2$*Lys*-Phe-Lys-IleNH$_2$ (45.7 mg, 43%). MS (ESI+) calc for $C_{87}H_{135}N_{15}O_{24}$ [M]$^+$: 1773.98, found 1774.8.

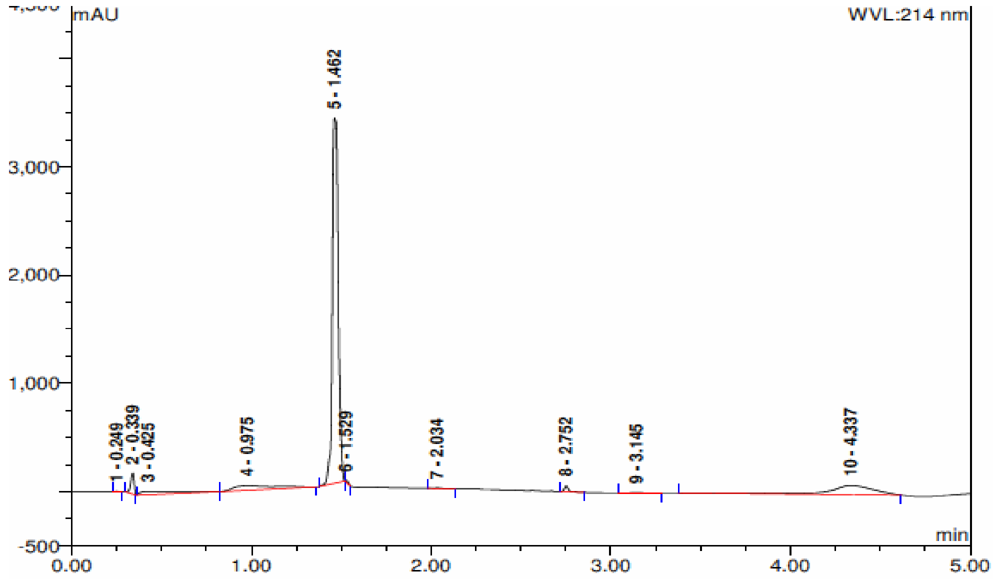

Figure S10. Analytical RP-UHPLC of **GalAG1.**

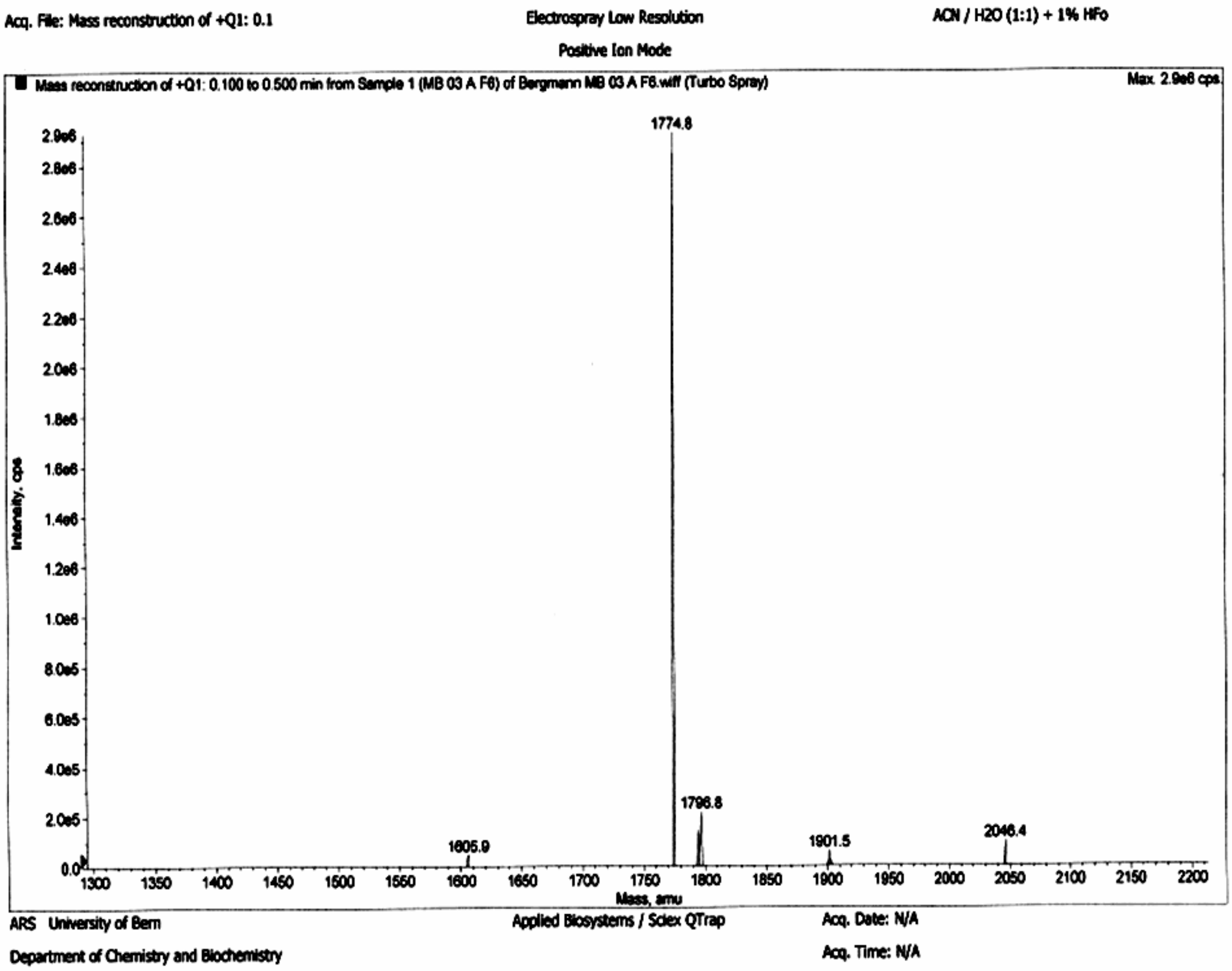

Figure S11. ESI-MS of **GalAG1.**

GalBG1: (GalB-Lys-Pro-Leu)$_2$*Lys*-Phe-Lys-IleNH$_2$ (39.5 mg, 39 %). MS (ESI+) calc for $C_{79}H_{135}N_{15}O_{22}S$ $[M]^+$: 1709.93, found 1710.8.

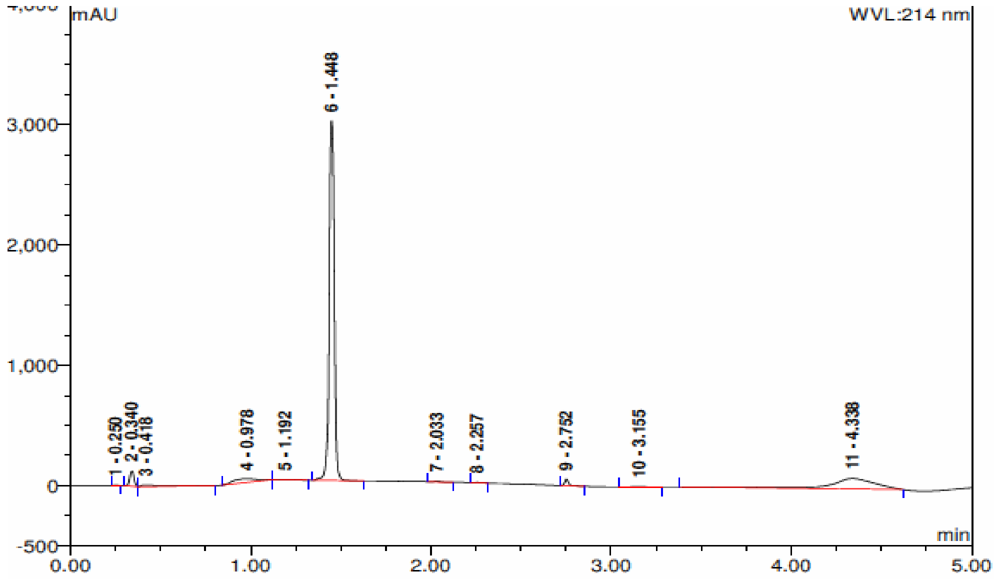

Figure S12. Analytical RP-UHPLC of **GalBG1.**

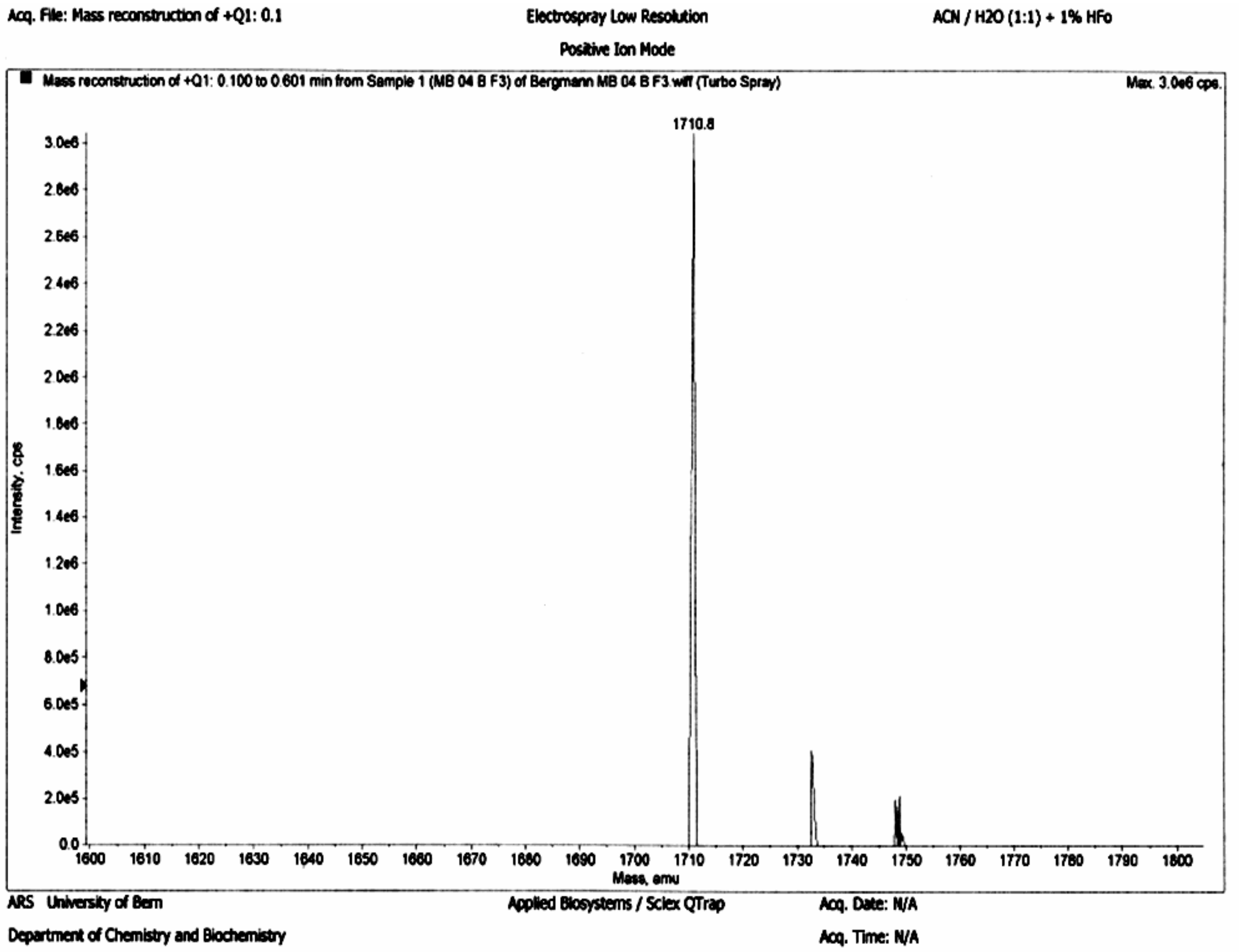

Figure S13. ESI-MS of **GalBG1.**

GalBG2: (GalB-Lys-Pro-Leu)$_4$(*Lys*-Phe-Lys-Ile)$_2$*Lys*-His-IleNH$_2$ (22.1 mg, 10 %). MS (ESI+) calc for $C_{176}H_{296}N_{35}O_{47}S_4$ $[M+H]^+$: 3783.08, found 3783.

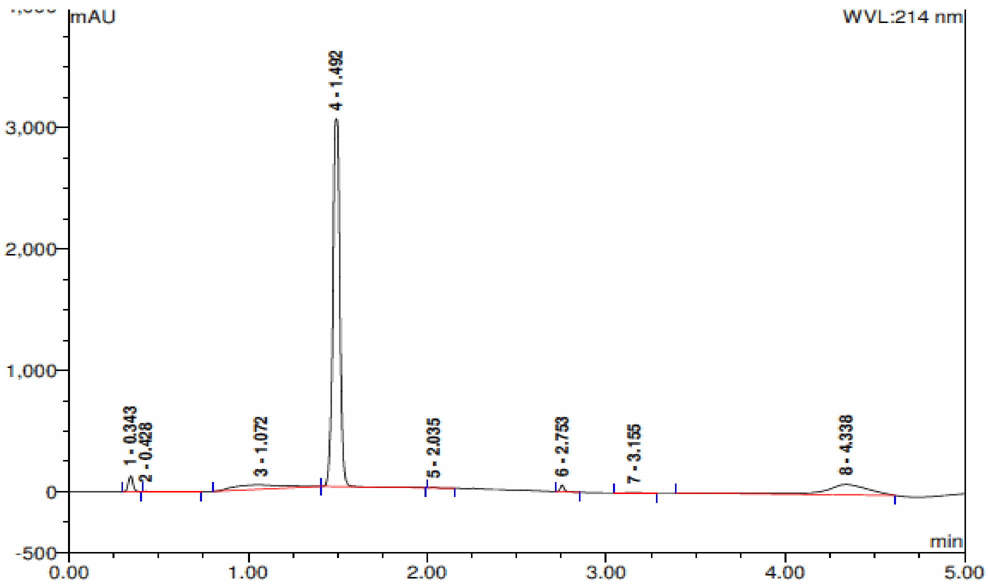

Figure S14. Analytical RP-UHPLC of **GalBG2.**

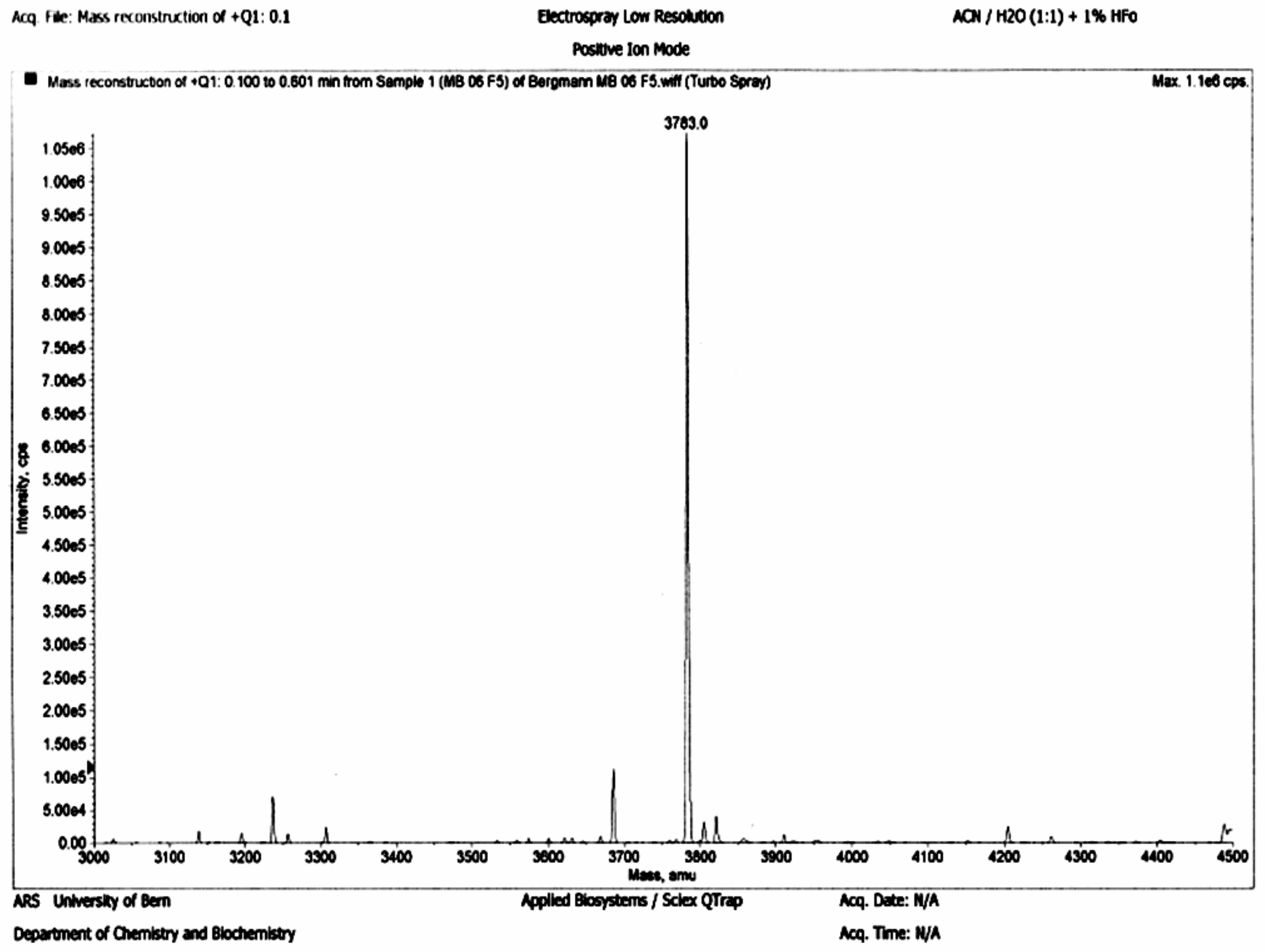

Figure S15. ESI-MS of **GalBG2.**

GalAG2: (GalA-Lys-Pro-Leu)$_4$(*Lys*-Phe-Lys-Ile)$_2$*Lys*-His-IleNH$_2$ (18.2 mg, 7%). MS (ESI+) calc for $C_{192}H_{297}N_{35}O_{51}$ [M+H]$^+$: 3909.17, found 3911.

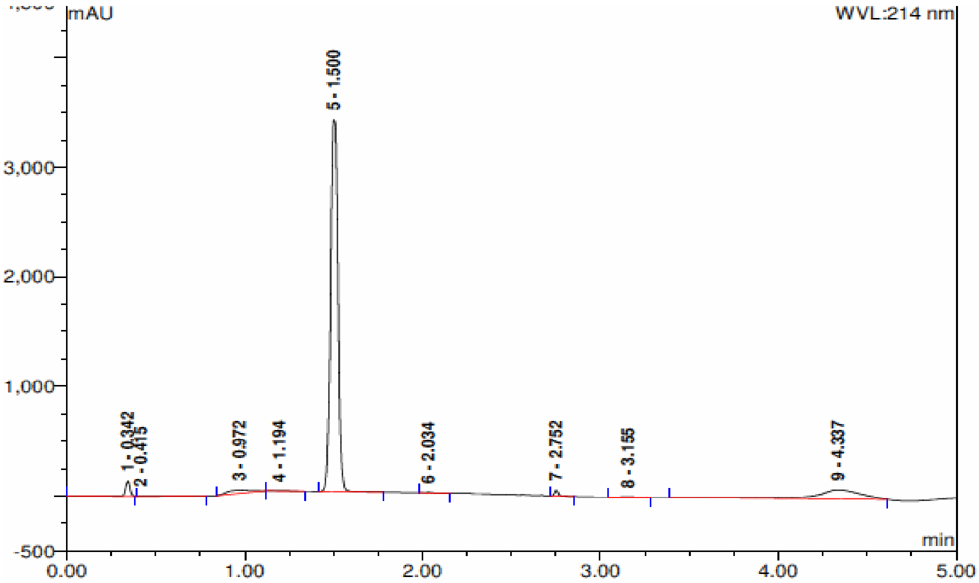

Figure S16. Analytical RP-UHPLC of **GalAG2.**

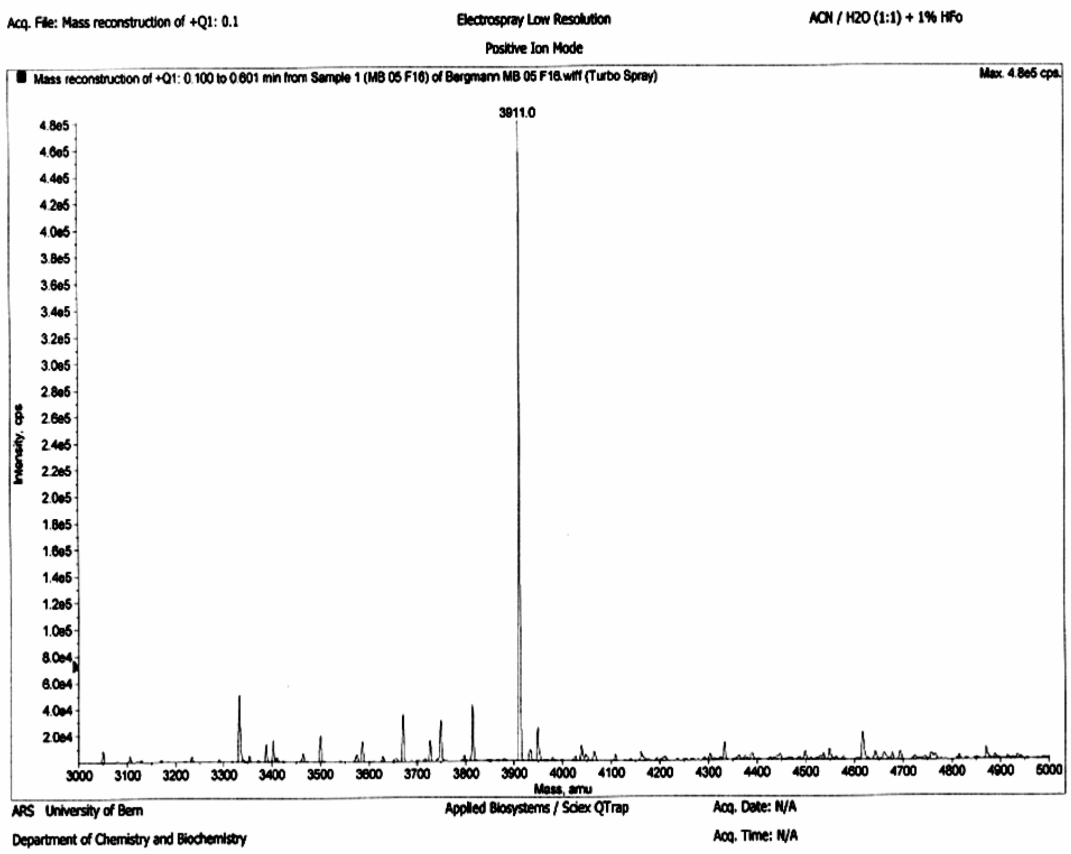

Figure S17. ESI-MS of **GalAG2.**

Acetylated G2 (AcG2): (Ac-Lys-Pro-Leu)$_4$(*Lys*-Phe-Lys-Ile)$_2$*Lys*-His-IleNH$_2$ (46.2 mg, 21%). MS (ESI+) calc for $C_{148}H_{249}N_{35}O_{27}$ [M+H]$^+$: 2950.8, found 2951.

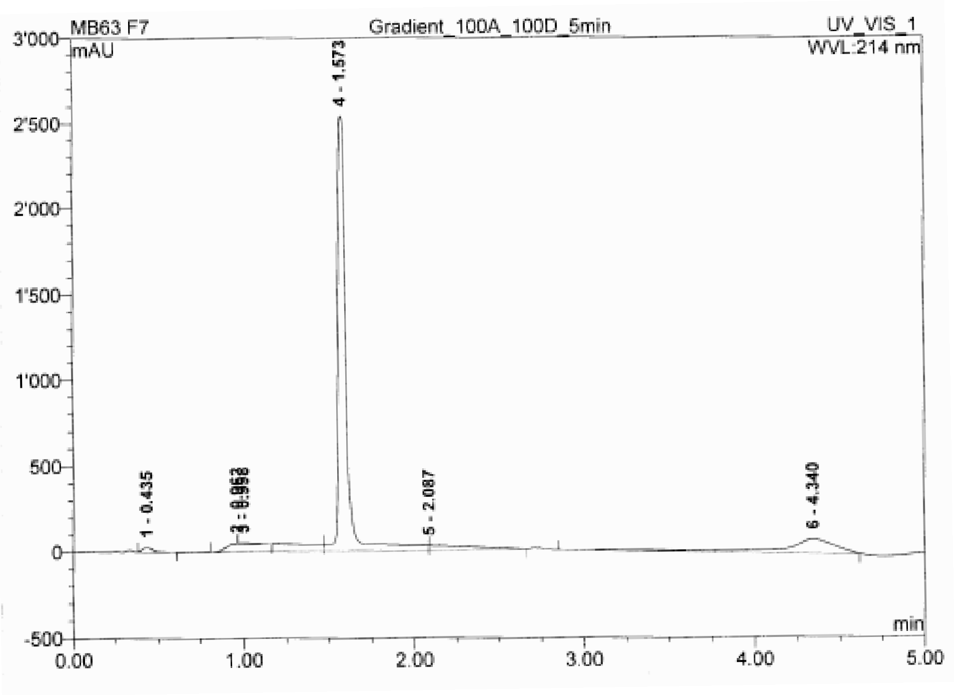

Figure S18. Analytical RP-UHPLC of **AcG2.**

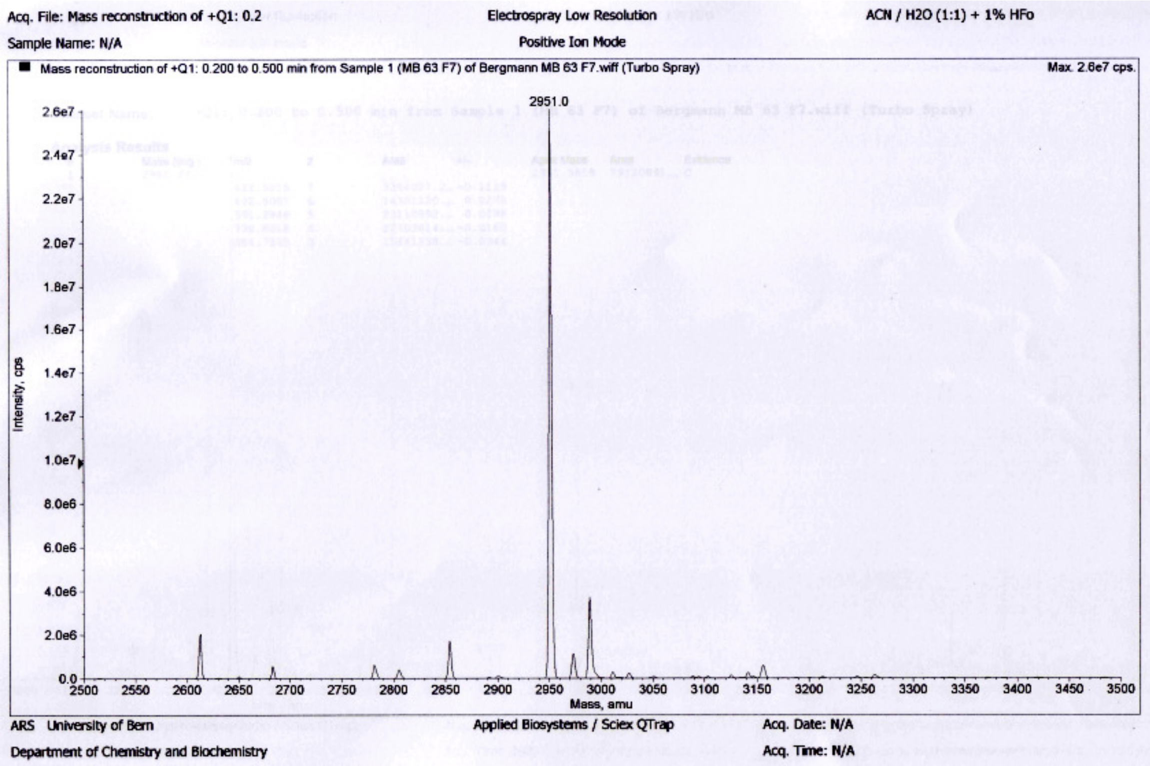

Figure S19. ESI-MS of **AcG2.**

Biofilm Assays

Incubation

The method is adapted from the original method by Dhir & Dodd[1] and similar to the related paper by Johansson et al.[2] Biofilms were grown on steel coupons (316L) in petri dishes containing 20ml 0.05% (v/v) NB2 (nutrient broth number 2) media and the appropriate concentration of the test dendrimer. Plates were inoculated with 200ul of a standard overnight inoculum of PAO1 grown in Luria Bertani (LB) media at 37°C with shaking at 200 rpm and previously washed twice in PBS. Coupons were incubated at 37oC on a rotary shaker at 60rpm. After 24 hours 200µl LB media was added and incubation continued under the same conditions for a further 24 hours. The supernatant was removed and the coupons were washed twice in 20ml PBS and once in 20ml of water, with gentle agitation for five minutes for each wash.

Staining

Two hundred microlitres of acridine orange (0.1% w/v) were added to the steel coupon in order to stain the remaining attached cells. This was left for 2 minutes 30 seconds. Excess acridine orange was removed by the addition of 20ml sterile PBS and the dish agitated for 5 minutes and the waste removed. This step was repeated once further with sterile PBS and once with sterile distilled water and left to air dry. The coupons were air dried and mounted onto a microscope slide.

Imaging and analysis

The coupons were placed in the universal slide holder of an inverted Zeiss LSM 700 laser scanning confocal microscope (Carl Zeiss, Germany).

Surface attachment. Coupons were imaged for bacterial attachment using the x10 objective lens, 405nm laser with pinhole of 94.2nm, gain set at 708 and digital offset at -1. Six images were taken at random from each coupon. The images were uploaded to ImageJ (NIH, USA) and thresholded between 40 and 164. The area fraction was calculated and the average surface area and standard error was calculated.

Biomass. Coupons were imaged for biomass using the x40 objective lens, 405nm laser, pinhole 73.3, gain set at 722, digital offset -43. Z-stacks were captured with averaging of 4 for each slice. Stacks were analysed using the COMSTAT2 (www.comstat.dk) plugin in ImageJ (NIH, USA). Images were subject to thresholding at 126 and biomass calculated with connected volume filtering active, without smacking. The results were exported to excel and standardised to the activity of the wild-type PAO1 strain. The results were exported to GraphPad Prism 5 for analysis.

P. aeruginosa lectin LecA expression and purification

LecA was expressed and purified by affinity chromatography along an optimized protocol and in accordance to a previous report.[3] The plasmid pET25paIL was transformed into *Escherichia* coli BL21(DE3) cells. *E.* coli cells were grown in 6L of LB medium (10gm tryptone; 5gm yeast extract and 5gm Nacl in 1L of deionised water) at 30 °C. When the culture had reached an optical density of 0.5–0.6 at 600 nm, isopropyl-β-D-thiogalactopyranoside (IPTG) was added to a final concentration of 0.1 mM. Cells were harvested after overnight shaking at 220 rpm at 20 °C, washed, and resuspended in 100 ml of loading buffer (20 mM Tris–HCl and 100 μM $CaCl_2$, pH 7.5). The cells were broken by sonication. After centrifugation at 5000 RPM for 45 min the supernatant was loaded to an affinity chromatography column containing 250 ml of Sepharose 4B (SI Figure S20). LecA was eluted with 0.2 M D-galactose in buffer (20 mM Tris–HCl and 100 μM $CaCl_2$, pH 7.5). The purified protein was extensively dialyzed against distilled water containing 2 μM $CaCl_2$ for 7 days and characterized using SDS-page and mass spectroscopy respectively (SI Figures, S21 and S22). Purified fractions of protein were lyophilized, and kept at −20 °C.

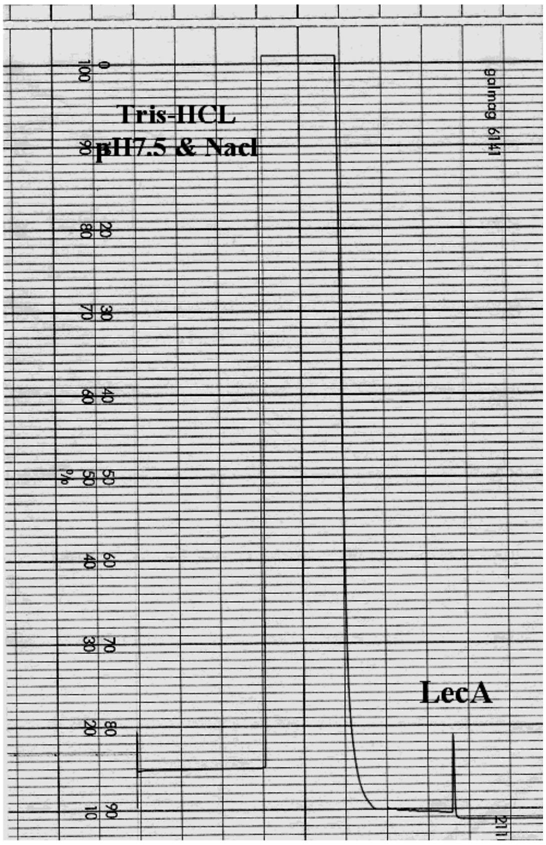

Figure S20: LecA purified using affinity chromatography on Sepharose 4B (GE healthcare). LecA was eluted with 0.2M D-galactose in buffer (20 mM Tris–HCl and 100 μM $CaCl_2$, pH 7.5).

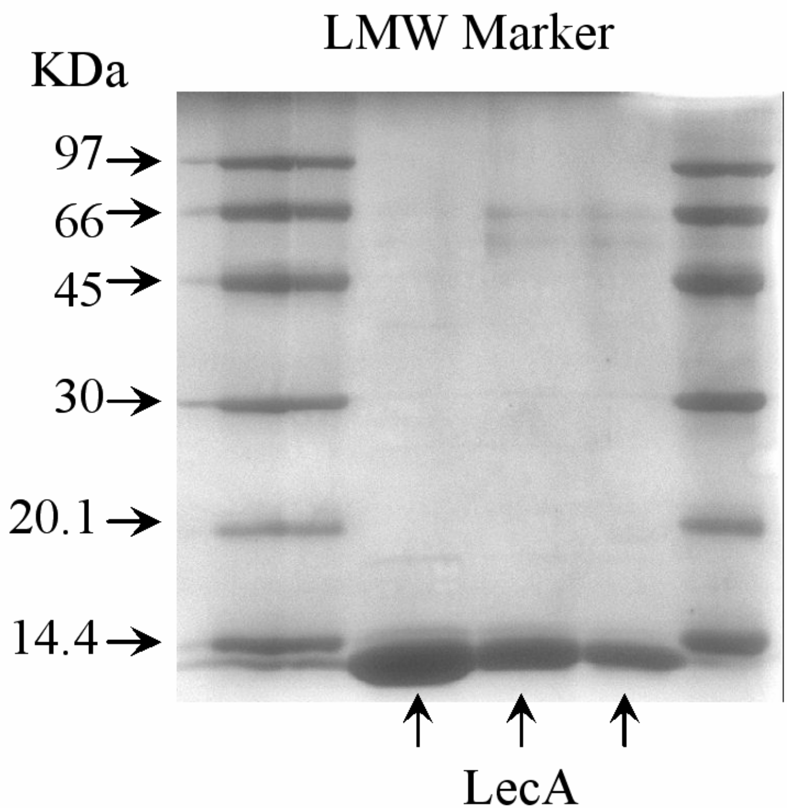

Figure S21: SDS-page analysis of expressed and purified LecA lectin.

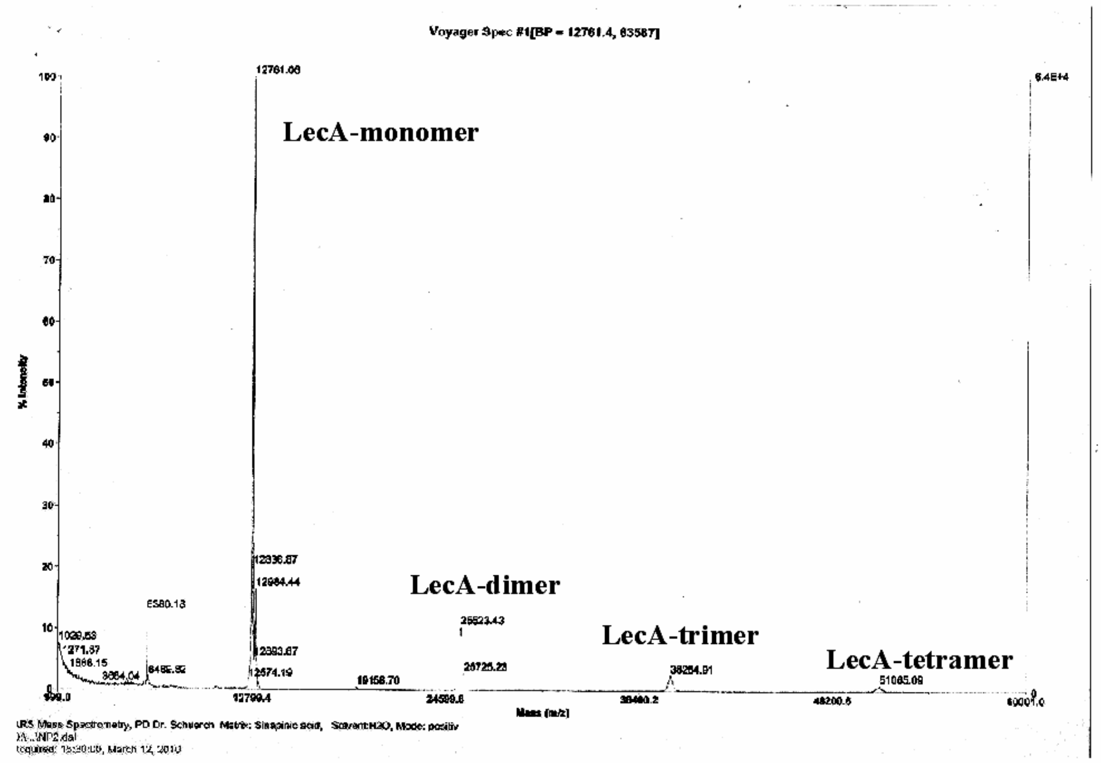

Figure S22: Determination of the precise molecular masses of purified LecA using MALDI-TOF mass spectrometry. The purified lectin preparations (giving a single band in gel electrophoresis) were used. Mass spectrometry was performed by the using a Matrix-assisted laser desorption ionization time of flight (MALDI-TOF) mass spectrometer.

Hemagglutination Assay

A) Erythrocytes preparation. Rabbit red cells (erythrocytes 50%; Biomerieux) separated from preservative bye centrifugation (1500 RPM; 10min), were washed three times with 0.9% Nacl solution (saline) and suspended to a concentration of 5% v/v in phosphate buffer saline (PBS; 0.01 M; pH 7.4). The suspension was given papain-treatment which includes incubation of 9 volumes of the 5% cell suspension with 1 volume of the 1% w/v papain (crude preparation, Sigma) in 0.1% w/v L-cysteine solution at 37˚ for 30 min. The enzyme treated cells were washed three times in PBS and then resuspended in it to a concentration of 5%.

B) LecA titration. In order to determine the lectin concentration needed to agglutinate the cells, decreasing amounts of LecA were incubated with red blood cells. Serial two fold dilutions were made in the wells of microtiter plate (96-well microtiter non-treated V-bottom plates, Nunc, Denmark). The two fold dilutions were made by adding 50 µL of buffer solution to the all 24 wells and 50µL of LecA solution (0.84 mg/ml) to the first well. 50µL was then transferred from the first well to the second. The second well was mixed and 50 µL was transferred to the third well. This procedure was repeated until the 24th well. To each well 50 µL of the RBCs solution (5% in PBS) was added and incubated for 30 min at 4˚C. After this time, plates were centrifuged for 30s (1,000 x g), the wells were examined and the minimum amount of LecA required to agglutinate the cell suspension was determined. This was then considered to be 1 HA unit. For the inhibition assay an 8 HA unit LecA solution was made up (SI Figures, S23).

c) Minimum Inhibitory Concentration (MIC) determination. A 50 µL sample of each inhibitor examined was serially diluted with 50 µL PBS in the microtiter plate to produce twofold dilutions (as described above). The inhibitor solutions were incubated with 50 µL of the 8 HA unit LecA solution (Conc. of LecA = 13.12 µg/mL) for 30 min at 4˚C. After this time 50 µL of the erythrocytes in PBS suspensions (Conc.5%) was added and the wells were mixed and incubated for one hour in the room temperature The plates were then centrifuged for 30s (1,000 x g). Each test was performed in triplicate. The activity of the tested compounds was recorded as minimal inhibitory concentration (MIC), corresponding to the highest dilution causing a complete inhibition of hemagglutination (SI Figures, S24).

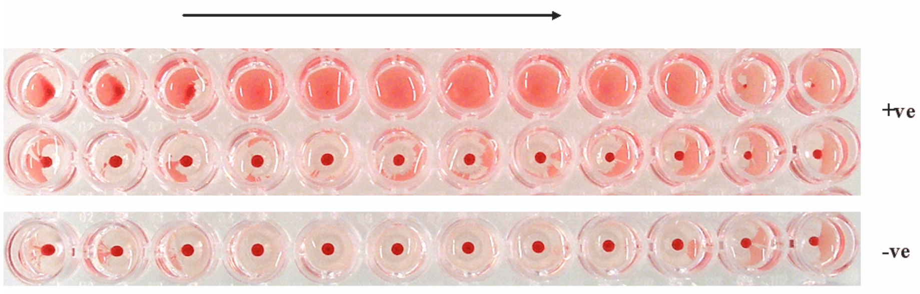

Figure S23. Lectin LecA titrations: hemagglutination assay with twofold serial dilutions of 50 µL of LecA solution (conc. 840 µg to 0.1 ng of per well, arrow) with 50µL of rabbit erythrocytes(Conc. 5% in PBS) was added and incubated for 30 min at 4°C and the amount of LecA required to agglutinate the cell suspension was determined (one HA unit; corresponds to 10th well in '+' sign block). Rabbit erythrocytes with PBS in absence of LecA show no agglutination ('-' sign block).

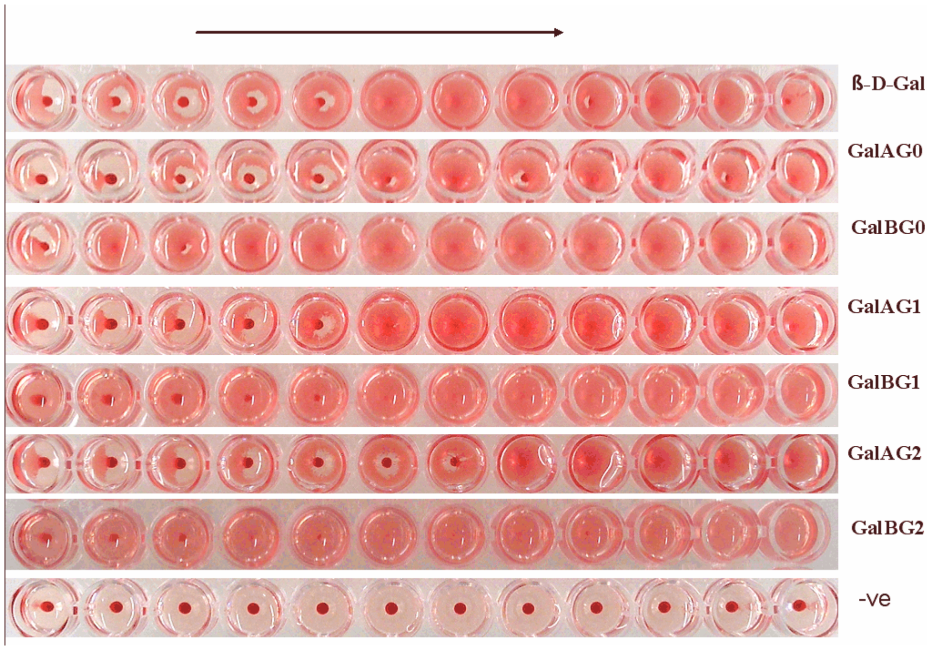

Figure S24. The effect of **GalA/B** dendrimers on receptor binding. Relative binding affinity of different dendrimers were assessed by hemagglutination assay with rabbit erythrocytes and twofold serial dilutions of each of the dendrimers (arrow; D-Galactose (25 mM); **GalAG0** (10 mM); **GalBG0** (10 mM); **GalAG1** (500 µM); **GalBG1** (5 mM); **GalAG2** (50 µM) and **GalBG1**(500 µM).

Isothermal Titration Calorimetry (ITC)

Lyophilized LecA was dissolved in buffer (0.1 M Tris-base, pH 7.5, 25 mM $CaCl_2$). Protein concentration was checked by measurement of absorbance at 280 nm using a theoretical molarity extinction coefficient of 27,600 $M^{-1}cm^{-1}$. Ligands were dissolved directly into the same buffer. ITC was performed with a iTC_{200} calorimeter (MicroCal Inc.). Titration was performed on 50-100 µM LecA in the 200 µl sample cell using 1-2 µl injections of 1-4 mM ligand every 180s at 25 °C. For reverse titrations performed on G1 and G2 of **GalA** and **GalB** series, LecA was taken in the syringe at concentrations ranging from the 0.25–1 mM and the ligand was taken in the cell at concentrations ranging from 5-40 µM. The data were fitted with MicroCal Origin 8 software, according to standard procedures using a single-site model. Change in free energy ΔG was calculated from the equation: $\Delta G = \Delta H - T\Delta S$ where T is the absolute temperature, ΔH and ΔS are the change in enthalpy and entropy respectively. Two independent titrations were performed for each ligand tested (Figure S25 and S26).

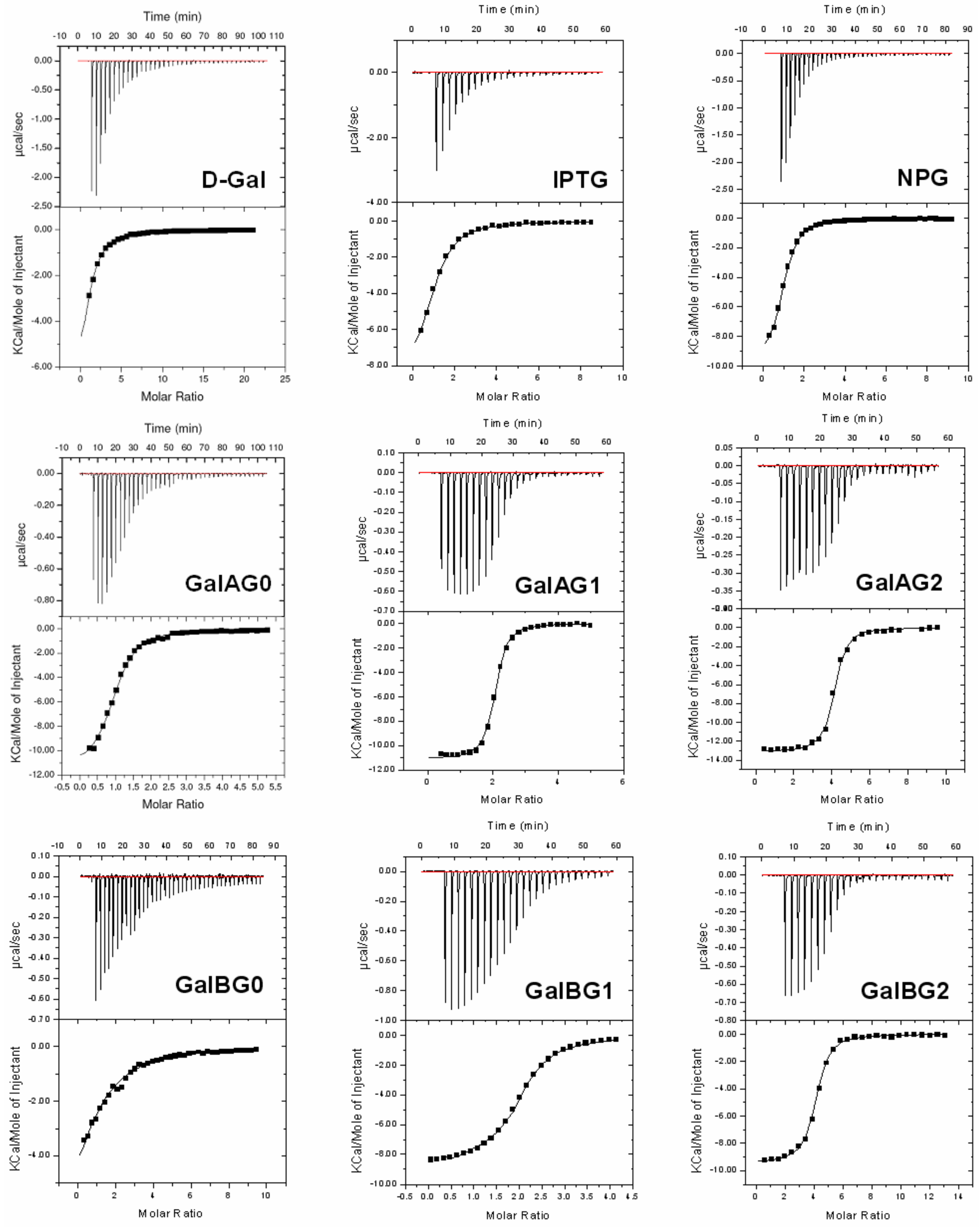

Figure S25. Isothermal Titration Calorimetric (ITC) measurements representing the raw ITC data (above) and integrated titration curves (below) for the binding of monovalent, divalent and tetravalent ligands to LecA. Titration type and corresponding concentrations for LecA/ligands are indicate in brackets (**D-Gal** (regular, 100 µM /10mM); **IPTG** (regular, 100 µM/4000 µM) and **NPG** (regular, 100 µM/4000 µM); **GalAG0**(regular, 40 µM/1000 µM); **GalAG1** (inverse, 470 µM/20 µM); **GalAG2** (inverse, 250 µM/5 µM); **GalBG0** (regular, 45 µM/2000 µM); **GalBG1** (inverse, 800 µM/40 µM); **GalBG2** (inverse, 400 µM/7 µM).

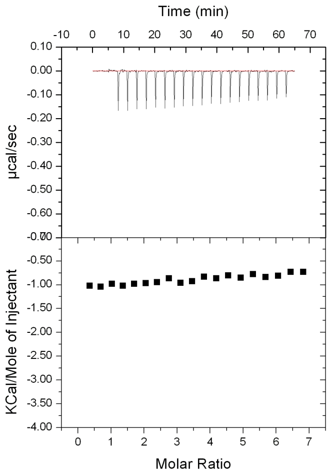

Figure S26. Isothermal Titration Calorimetric (ITC) measurement representing the raw ITC data (above) and integrated titration curves (below) for the binding of acetylated control dendrimer **AcG2** (2 mM) to LecA (50 µM). The Ac-Lys-Pro-Leu)$_4$ (*Lys*-Phe-Lys-Ile)$_2$ *Lys*-His-Ile-NH$_2$ dendrimer lacks sugar moieties and does not bind to LecA.

X-ray Crystallography

Co-crystallization of **NPG**, **GalAG0** and **GalBG0** with LecA lectin was carried out by the sitting drop method. In brief, lyophilized protein was dissolved in water (10 mg/ml) in the presence of salts (1 mM $CaCl_2$ and $MgCl_2$) and the respective galactoside ligand (0.5 mg/ml). In general crystals were obtained within three days after mixing 2 μL of LecA solution with 2 μL of reservoir solution at 20˚C (Figure S27). Primary crystallization conditions included screens I/II, Index I/II and SaltRx I/II respectively from Hampton Research (Laguna Niguel, CA, USA). Nicely diffracting crystals were found in screen I-33 (4.0 M Sodium formate); Index I-40 (0.1 M Citric acid pH 3.5 and 25% w/v Polyethylene glycol 3,350) and SaltRx II-16 (1.5 M Ammonium sulfate and 0.1 M Sodium acetate trihydrate pH 4.6) respectively.

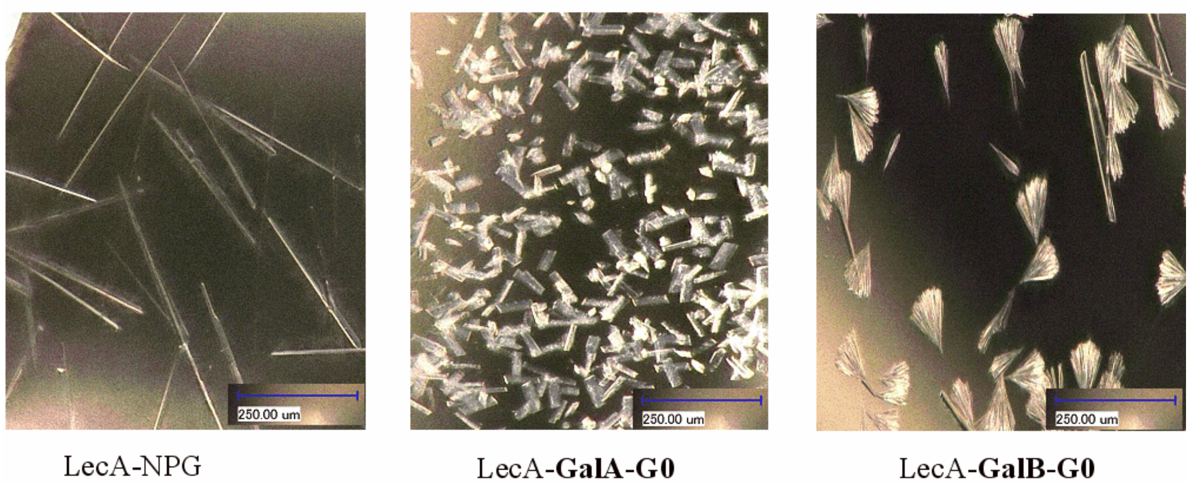

Figure S27. Crystals space for LecA in presence of different ligands: A) LecA-**NPG** B) LecA-**GalA-G0 and** C) LecA-**GalB-G0** crystal screen I-33 (4.0 M Sodium formate); Index I-40 (0.1 M Citric acid pH 3.5 and 25% w/v Polyethylene glycol 3,350) and SaltRx (1.5 M Ammonium sulfate and 0.1 M Sodium acetate trihydrate pH 4.6)

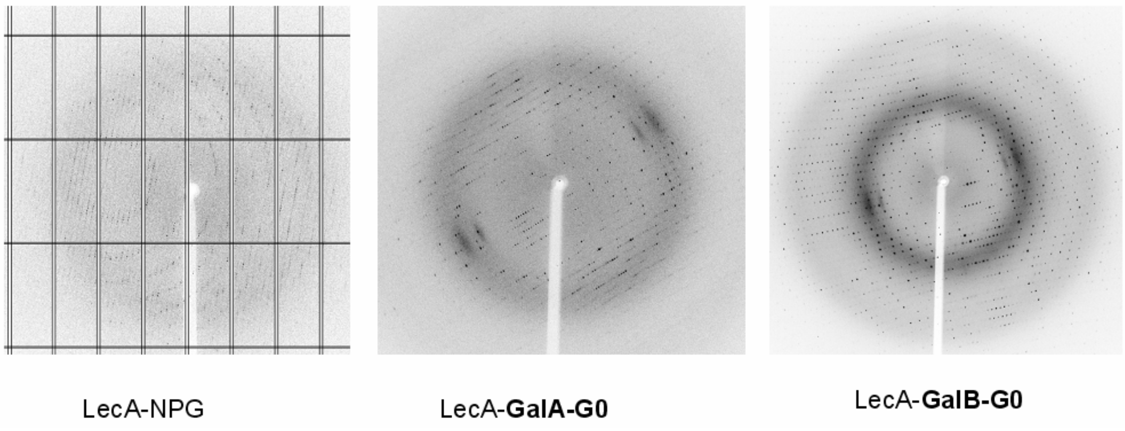

Figure S28. Diffraction pattern of co-crystallized LecA with different liagnds. A) LecA-**NPG** B) LecA-**GalAG0** and C) LecA-**GalBG0**

LecA-galactoside crystals belong to space groups $P4_12_12$, $P2_12_12_1$ and $P2_12_12$ with the corresponding asymmetric units containing four, eight and two monomers for **NPG**, **GalAG0** and **GalBG0** respectively. Further details on data collection statistics are given in (Table S1). Crystals were cryo-cooled at 100 K after soaking them for as short a time as possible in glycerol 25% v/v in precipitant solution. All data were collected at the SLS synchrotron (Villigen, Switzerland) at beamline PX-II/III (Figure S28).

Table S1. Data collection and refinement statistics for the **GalAG0. LecA GalBG0. LecA** and **NPG. LecA**

Structural data	GalAG0. LecA	GalBG0. LecA	NPG. LecA
Beam line	PSI PX III	PSI PX III	PSI PX II
Wavelength (Å)	1.00000	1.00000	1.00000
Resolution (Å)	73.07-2.29 (2.43-2.29)*	66.86-1.50 (1.59-1.50)*	81.86-1.94 (2.06-1.94)*
**Cell dimension**			
Space group	$P2_12_12_1$	$P2_12_12$	$P4_12_12$
Unit cell (Å)	a = 53.4, b = 128.6, c = 146.1; $\alpha = \beta = \gamma = 90°$	a = 40.6, b = 72.9, c = 133.7; $\alpha = \beta = \gamma = 90°$	a = 84.6, b = 84.6, c = 163.7; $\alpha = \beta = \gamma = 90°$
Measured reflection / unique	164993 / 45434	215327 / 61876	284443 / 44306
Average multiplicity	3.6 (3.5)	3.5 (2.9)	6.4 (6.5)
Completeness (%)	98.8 (93.5)	96.1 (84.2)	99.5 (97.4)
Average $I / \sigma(I)$	10.2 (2.8)	34.5 (14.8)	12.2 (3.5)
R_{sym} (%)	10.2 (44.2)	2.7 (6.6)	9.2 (38.8)
Wilson B-factor	30.0	16.8	33.6
**Refinement**			
Resolution range (Å)	49.26-2.29	49.26-1.50	48.28-1.94
R_{work} (%)	21.37	18.55	18.55
R_{free} (%)	24.04	20.15	21.00
Average Biso (Å^2)			
All atoms	26.19	14.94	31.20
Protein atoms	25.91	10.03	30.41
Sugar atoms	27.22	10.36	35.53
Solvent atoms	28.42	28.99	39.06
RMSD from ideality angles (°)	0.691	1.108	0.722
Bonds (Å)	0.002	0.005	0.003
Water molecules	945	646	391
Number of galactose	8	2	4
Calcium atoms	8	2	4
Protein Data Bank deposition code	3zyb	3zyh	3zyf

*Values between parentheses correspond to the highest resolution shell

Data were integrated and scaled with the X-ray detector software for processing single-crystal monochromatic diffraction data (XDS).[4] The structures of the co-crystallized ligands were solved by the molecular replacement technique with the Phaser program[5], using the monomeric structure (PDB code 1OKO)[6] of the calcium and galactose containing LecA with galactose, calcium and water molecules removed from the search probe. The molecular replacements gave clear solutions for all three ligand complexes and the corresponding electron density maps of the complexes showed clear features corresponding to the respective ligand. Automatic placement of water molecules was performed using the ARP/wARP program[7]. Crystallographic refinements were carried out with the program phenix.refine from the PHENIX program package[8] and manual model building with COOT[9].

All figures were generated by using the program PYMOL (www.pyMol.org). The electrostatics were calculated using the PDB2PQR server and the Adaptive Poisson-Boltzmann Solver software for continuum electrostatics calculations using a protein dielectric of 2.0, a solvent dielectric of 80, an ion exclusion radius of 2.0 Å, a probe radius of 1.4 Å, and an ionic strength of 0.14 M[10].

Ligand induced conformational changes in LecA

The T-shaped interaction mode between **NPG** and His50 within the LecA-**NPG** binding site provides a positional anchor for further extensions of the ligand as found in the **GalAG0**.LecA complex. The tripeptide moiety of **GalAG0** adopts conformations complementary to the protein surface (Figure S29). Consequently, three distinct conformations of the tripeptide moiety within the **GalAG0**.LecA crystal structure were observed (Figure S29 and S30).

Previous experimental analysis using binding assays and thermodynamics of **NPG**.LecA, **GalAG0**.LecA and **GalBG0**.LecA show that **GalAG0** displays 22 fold increase in binding affinity compared to galactose and 4 fold compared to **NPG**. We propose that preferential binding of the **GalAG0** towards LecA is due to strong edge-to-face π-π interactions and is further enhanced by conformational complementarity available to the peptide portion. The comparative analysis of our co-crystal structures with apo LecA (PDB code-1UOJ)[18] reveals that **GalAG0** induces conformational changes in the loop region from residues 40-55 resulting in hydrogen bonding between Gln53 and the Lysine side-chain of **GalAG0**. The Calcium Recognition Domain (CRD) region mostly remains unaltered except for slight conformational changes of the side chains of Asn107 and Lys41 (Figure S31A). The overlay of the crystal structure of apo LecA with the one of **GalAG0**.LecA complex uncovers minor ligand induced shifts at the overall protein surface (Figure S31B).

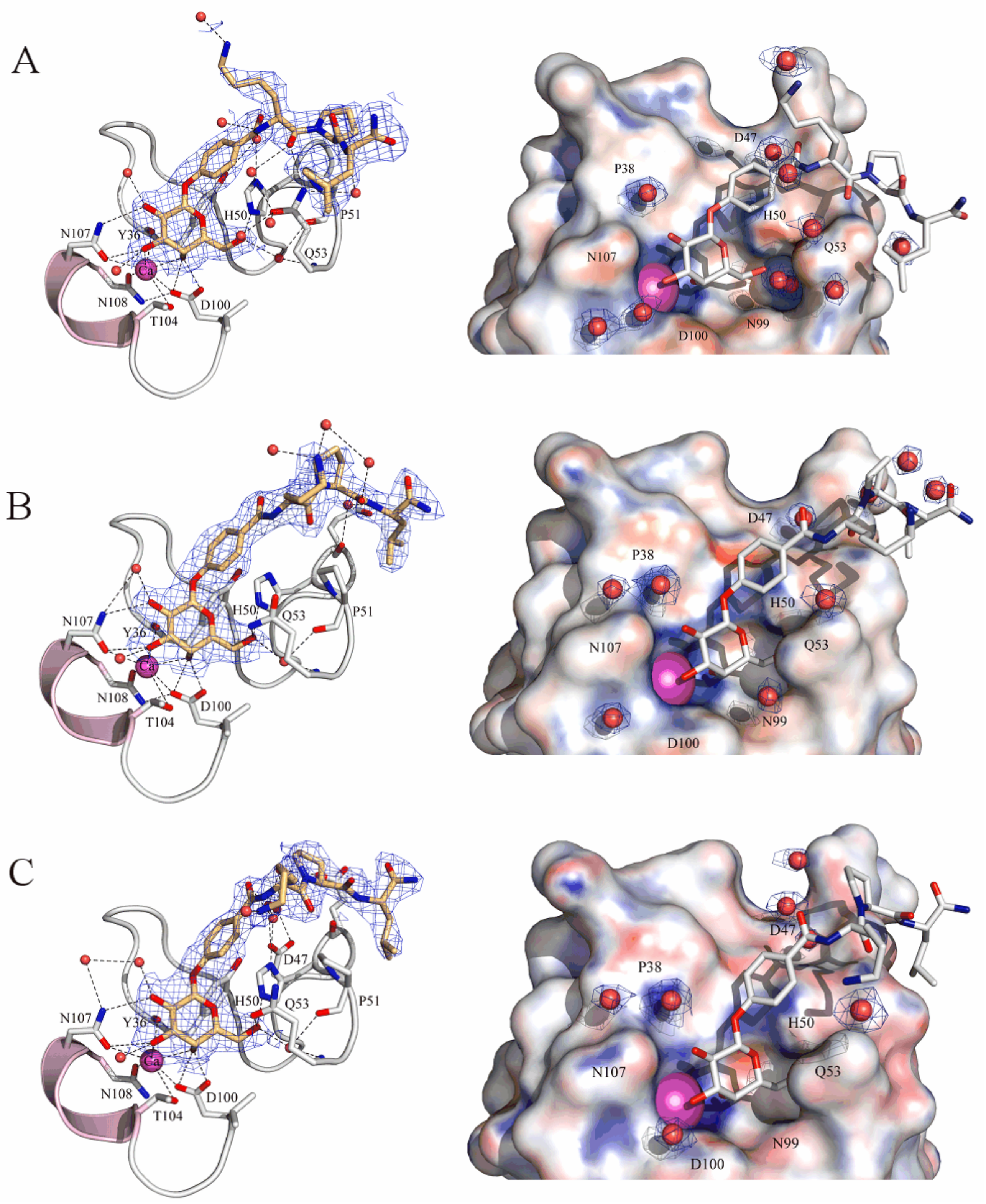

Figure S29. Electron density map (contoured at 1 σ level) around the **GalAG0**.LecA -complex A) ChainA, B) ChainB and C) ChainF. Electrostatic surface potentials ranging from -2 kcal/mol (red) to +2 kcal/mol (blue) were calculated using the PDB2PQR server and by the Adaptive Poisson-Boltzmann Solver software for continuum electrostatics calculations with a protein dielectric of 2.0, a solvent dielectric of 80, an ion exclusion radius of 2.0 Å, a probe radius of 1.4 Å, and an ionic strength of 0.14 M.

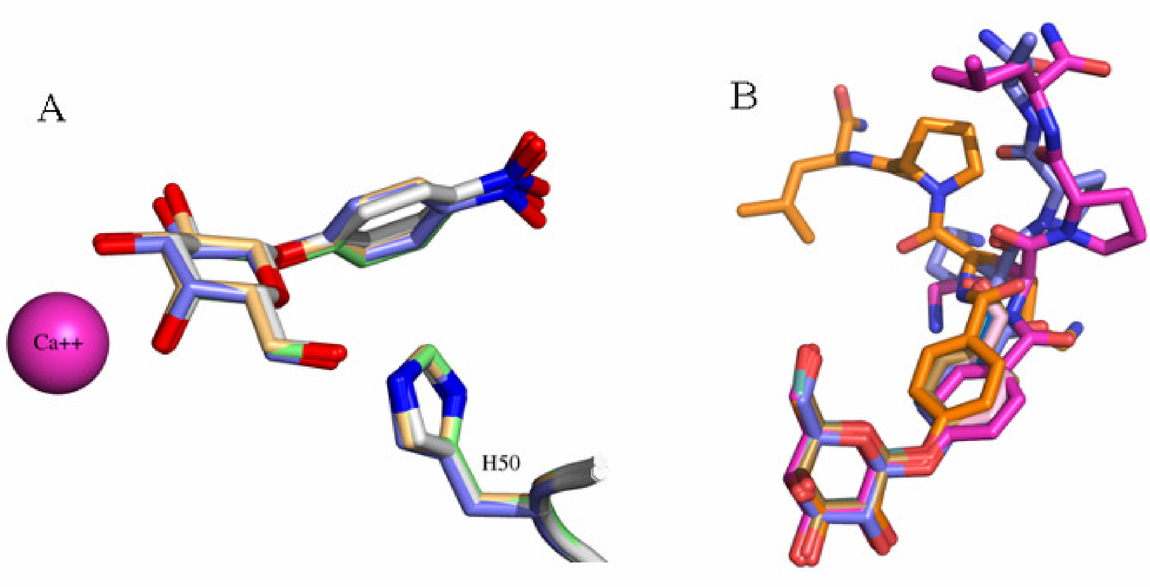

Figure S30. A) Superposition of the four subunits of the **NPG**.LecA -complex revealing minor alterations of the "T-Shape" interaction between LecA and **NPG**. Ca^{2+} ion represented as magenta sphere. B) Superposition of eight distinct conformations of the **GalAG0** ligand observed in LecA subunits A-H in the crystal structure. The phenyl moieties of the galactoside are in similar positions, while the tripeptide portion residues adopt distinct conformations.

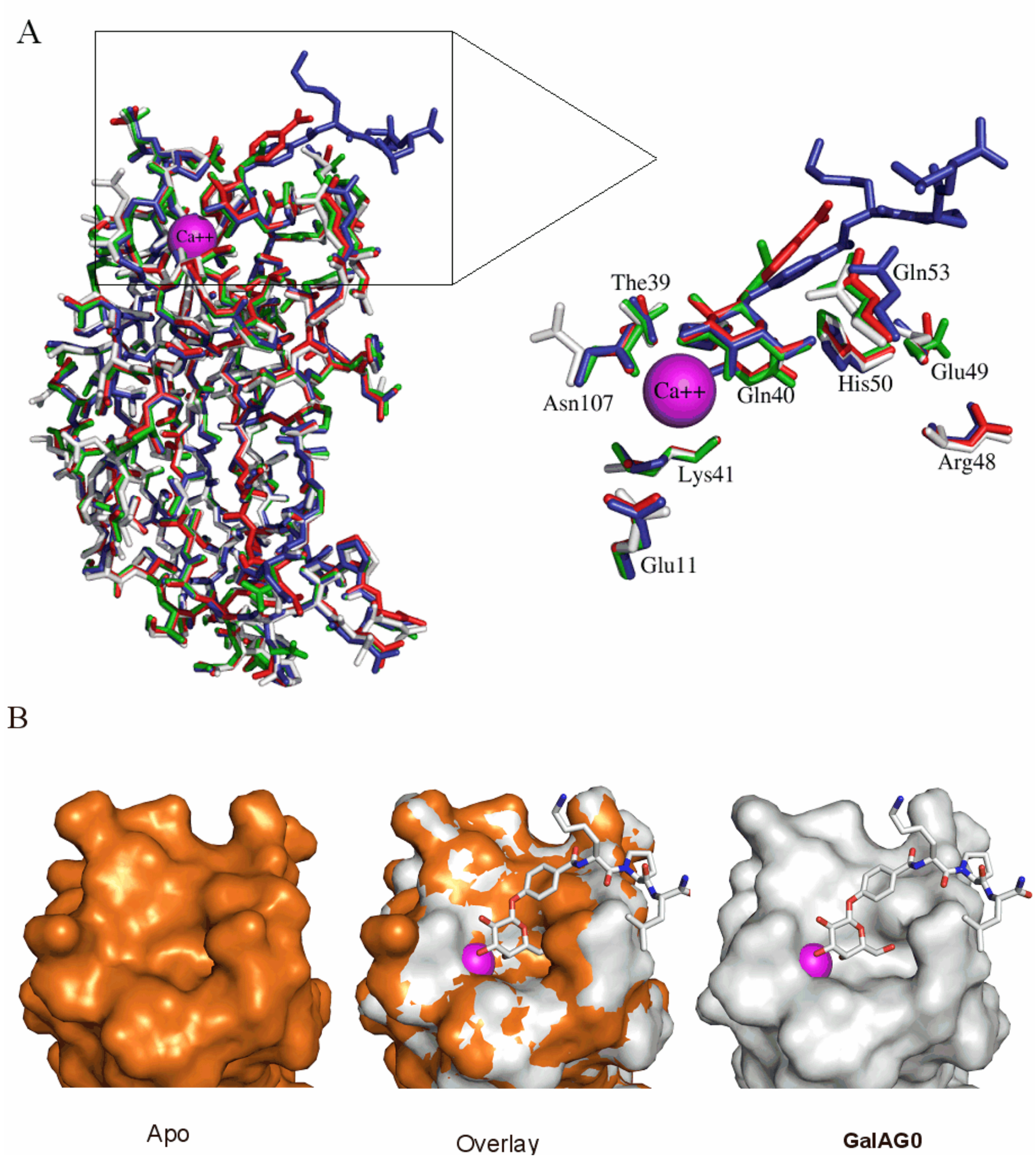

Figure S31. Ligand induced conformational changes in LecA. A) Overlay of apo LecA (white); **NPG** (red); **GalAG0** (blue) and **GalBG0** (green) with close up of the conformational changes within the loop region (Gln40-Gln53). B) Surface overlay of apo- LecA (PDB code 1UOJ ; orange color) with **GalAG0**- LecA (grey color).

Molecular Dynamic Simulations

Glycopeptide dendrimer structure building. Based on circular dichroism and FT-IR analysis showing 20-30% α-helix in **GalAG2** and **GalBG2** dendrimers (data not shown), the starting structures for free dendrimers in the present study were built with L-amino acids ordered as α-helix (figure S32), using peptide building dictionary interface in maestro version 8.5 in Schrödinger suite. Parameters for branching lysine residue were obtained from natural amino acid building block based on transferability of OPLS-AA (Optimized Potentials for Liquid Simulations-All Atom)[11] force field.

Macromodel Energy Optimizations. Built free dendrimers were optimized by checking for correct bond orders and performing H-bond treatment using default protocol from protein preparation wizard in maestro 8.5. The structures were subsequently minimized in macromodel (version 9.6) using steepest descent method with maximum of 500 iterations with gradient convergence threshold of 0.05 and constant dielectric with dielectric constant of 1. The potential force field used for minimizations protocol was OPLS-AA with extended cutoff values (van der Waals = 8.0Å; electrostatic = 20.0 Å; H-bond = 4.0 Å).

System Preparation for Simulations. Molecular dynamics simulations were performed using OPLS-AA force field in desmond molecular dynamics package with maestro-desmond interoperability tool, version 2.0[12] The systems were setup for explicit solvent simulations. The solute was immersed in SPC water model[13] in cubic box spaced at 1 nm from solute boundaries. The physiological salt concentration of 0.15 M was used.

Molecular dynamic simulations. The prepared systems were used for molecular dynamics simulation. MD simulations for a period of 10 ns were performed using following three steps : 1) minimization 2) equilibration and 3) production run;

*Minimization***.** The setup systems were minimized to remove close contacts between solute-solvent molecules using the LBFGS method with maximum iterations of 200 cycles and convergence threshold of 1.0 Kcal mol^{-1} $Å^{-1}$. The step size was 0.005 ps and switch criteria was 25.0 Kcal mol^{-1} $Å^{-1}$. The short range interactions used cutoff radius of 9.0 Å, whereas the long range coulombic interactions were taken into account using smooth Particle Mesh Ewald (PME) with Ewald tolerance of 1e-09 Å.

Equilibration protocol. During equilibration 2000 steps of steepest descent minimization of water molecules and ions were performed to allow water molecules to assume a lower energetic geometry, while the solute was restrained with force constant of 50 kcal/mol $Å^2$. The resulting

systems were then subjected to 2000 steps of minimization with no restraints, reaching a root mean square gradient of 0.1 to assure the relaxation of the structures, followed by 12 ps of heating from 10 to 300 K in a constant volume ensemble with restraints on the solute heavy atoms (50 kcal/mol $Å^2$). This was followed by 12 ps of constant pressure unrestrained simulation at 300 K, where convergences of energies, temperature, pressure and density of the systems was monitored.

Production runs protocol. The output co-ordinates and velocities from the equilibrated run were subsequently used in productions runs over a period of 10 ns at 300 K using NPT ensemble. The bond lengths to hydrogens were constrained with a variant of the M-SHAKE algorithm[14]. To maintain 1 atm at 300 K in NPT ensemble, the system was coupled to a Martyna-Tobias-Klein barostat[15], with relaxation time of 2 ps with isotropic coupling style and a Nose-Hoover thermostat[16] (with relaxation time of 1 ps). Long-range electrostatic interactions were modeled using a Particle Mesh Ewald method [17] and a 64 × 64 × 64 Fourier space mesh with fifth-order B spline interpolation. The van der Waals (VDW) interactions and real space contributions to the electrostatics were truncated at 10 Å, and estimated the long-range VDW contributions to the energy and the pressure by assuming a homogeneous distribution of VDW spheres with dispersion coefficient 69.5 kcal/mol/Å. RESPA integrator [18] with steps of 2 fs was used for bonded and short-range non bonded interactions, and 6 fs for long-range electrostatics. During the course of simulations, quality checks of system were performed by monitoring total energy profile and backbone atom RMSD of structures in trajectories (Figure S33).

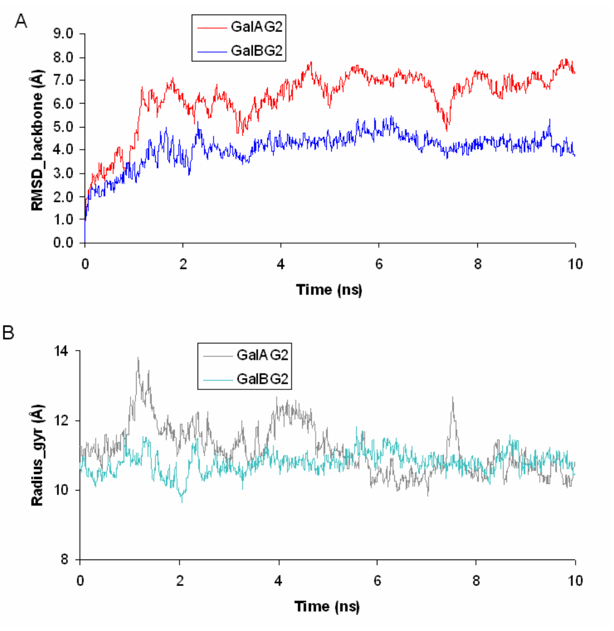

Figure S32 Plots for molecular dynamic simulation of **GalAG2** and **GalBG2** dendrimers; A) RMSD (backbone-backbone) and B) radius of gyration (R_g) as function of time.

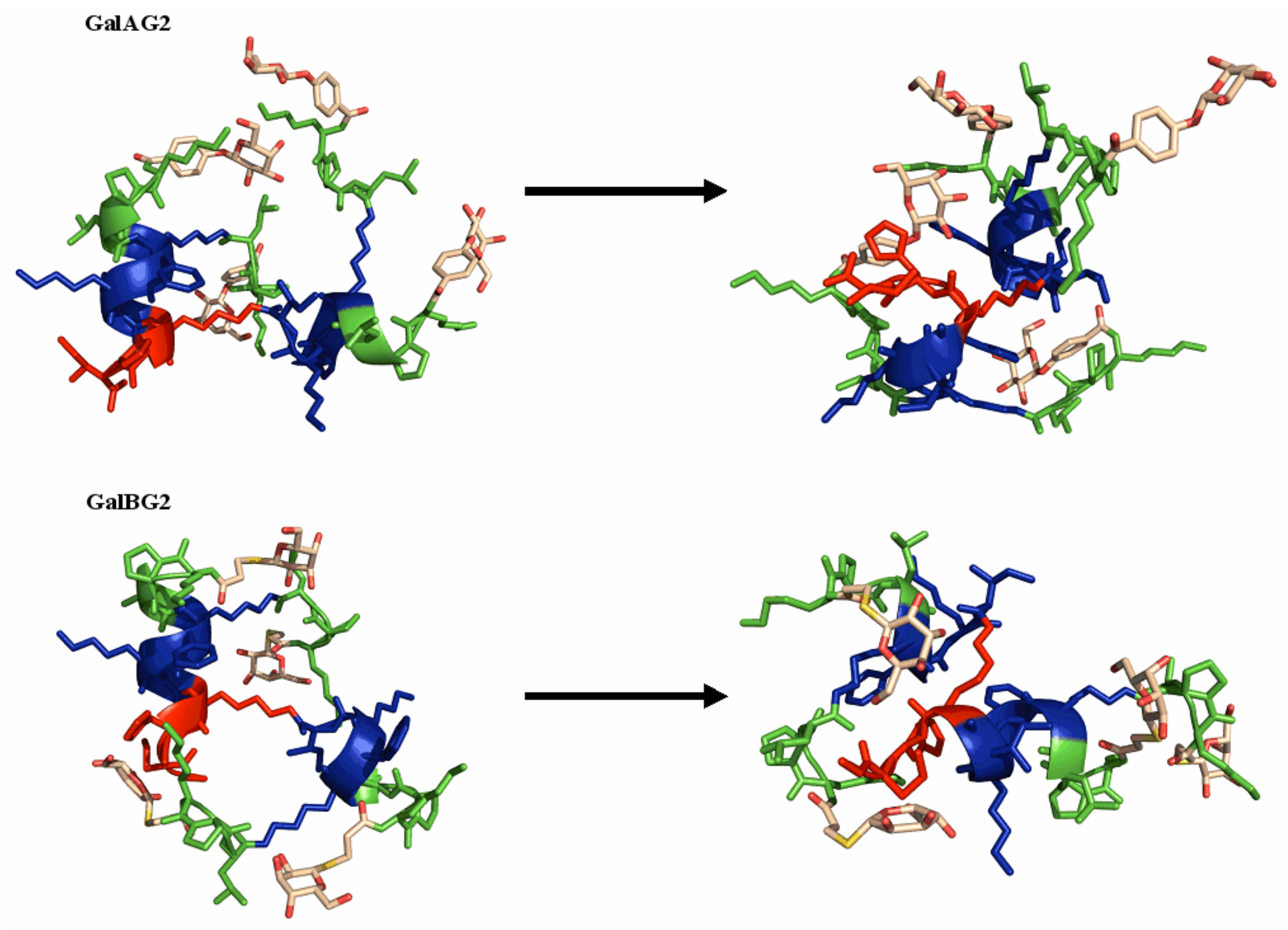

Figure S33. First and last snapshots from the molecular dynamics simulations of **GalAG2** and **GalBG2** dendrimers. Color codes indicates dendrimer generations, Red (G0); Blue (G1) and Green (G2).

Understanding the multi-valency effect in LecA–ligand complexes.

The final structures obtained from the molecular dynamics simulations of free **GalA/BG2** dendrimers were used as the starting conformation for modeling dendrimer.LecA complexes. In order to prepare the complexes, the terminal part that was observed in the crystal structures i.e. the terminal tripeptide segment fused to galactose via the phenyl linker in **GalA** and the galactose fused to the sulfur linker in **GalB**, was removed from one of the flanking chains of the modeled free dendrimer and the dendrimer fused to the corresponding **GalA** or **GalB** portion to form the **GalAG2.**LecA and **GalBG2.**LecA complexes respectively. The complexes were minimized to remove any steric hindrance and subsequently, simulated for 10 ns using same protocols as described for free dendrimers. During the course of simulation, **GalAG2**.LecA complex got stabilized within the first two ns, whereas, **GalBG2**.LecA complex showed more RMSD fluctuations (Figure 3A). Moreover at any given point in the simulation **GalAG2.**LecA complex showed about 14 intermolecular hydrogen bonds, whereas only 4 intermolecular hydrogen bonds existed in the **GalBG2**.LecA complex (Figure S34). These data suggest a higher stability for the **GalAG2.**LecA complex as compared to **GalBG2**.LecA complex and correlate well with X-ray crystallographic analysis where the **GalAG0**.LecA complex is better structured than **GalBG0**.LecA complex.

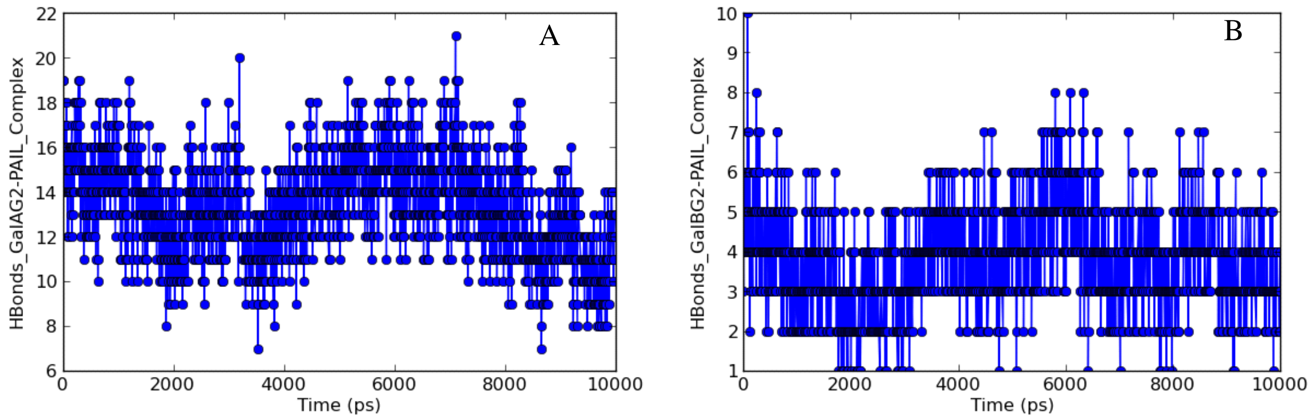

Figure S34 Intermolecular hydrogen bond analysis 10 ns trajectory of **GalAG2**.LecA complexes (A); **GalBG2**.LecA (B) complexes. Parameters used for defining H-bond- distance = 2.5Å; NH..X (donor angle) > 120° and H..X-Y (acceptor angle) > 90°

References

[1] V.K. Dhir, C.E .Dodd, *Appl. Environ. Microbiol.* **1995**, *61,* 1731–1738.

[2] E.M. Johansson, S.A. Crusz, E. Kolomiets, L. Buts, R.U. Kadam, M. Cacciarini, et al. *Chem. Biol.* **2008**, *15*, 1249-1257.

[3] B. Blanchard, A. Nurisso, E. Hollville, C. Tètaud, J. Wiels, M. Pokorna, M. Wimmerova, A. Varrot, A. Imberty, *J. Mol. Biol.* **2008**, *383*, 837-853.

[4] W. Kabsch, *Acta Crystallogr D Biol Crystallogr* **2010**, *66*, 125-132.

[5] McCoy, A. J.; Grosse-Kunstleve, R. W.; Adams, P. D.; Winn, M. D.; Storoni, L. C.; Read, R. J. *J Appl Crystallogr* **2007**, *40*, 658-674.

[6] G. Cioci, E. P. Mitchell, C. Gautier, M. Wimmerova, D. Sudakevitz, S. Perez, N. Gilboa-Garber, A. Imberty, *FEBS Lett* **2003**, *555*, 297-301.

[7] A. Perrakis, R. Morris V.S. Lamzin, *Nat Struct Biol* 1999, 6, 458–463.

[8] P. D. Adams, P. V. Afonine, G. Bunkoczi, V. B. Chen, I. W. Davis, N. Echols, J. J. Headd, L. W. Hung, G. J. Kapral, R. W. Grosse-Kunstleve, A. J. McCoy, N. W. Moriarty, R. Oeffner, R. J. Read, D. C. Richardson, J. S. Richardson, T. C. Terwilliger, P. H. Zwart, *Acta Crystallogr D Biol Crystallogr*, *66*, 213-221.

[9] P. Emsley, K. Cowtan, *Acta Crystallogr D Biol Crystallogr* **2004**, *60*, 2126-2132.

[10] N.A. Baker, D. Sept, S. Joseph, M.J. Holst, J.A. McCammon *Proc. Natl. Acad. Sci. USA*, **2001**, *98*, 10037-10041.

[11] W. L. Jorgensen, D. Maxwell, J. Tirado-Rives *J. Am. Chem. Soc*. **1996**, *118*, 11225-11236.

[12] J. B. Kevin, C. Edmond, X. Huafeng, O. D. Ron, P. E. Michael, A. G. Brent, L. K. John, K. Istvan, A. M. Mark, D. S. Federico, K. S. John, S. Yibing, E. S. David *Proceedings of the 2006 ACM/IEEE conference on Supercomputing*; ACM: Tampa, Florida, **2006**.

[13] (a) B. P. Roberts, G. Y. Krippner, M. J. Scanlon, D. K. Chalmers, *Macromolecules* **2009**, *42*, 2784-2794; (b) B. P. Roberts, M. J.Scanlon, G. Y. Krippner, D. K. Chalmers, *Macromolecules* **2009**, *42*, 2775-2783.

[14] V. Krautler, W. F.Van Gunsteren, P. H. Hunenberger, *J. Comp. Chem*. **2001**, 22, 501–508.

[15] G. J. Martyna, D. J. Tobias, and M. L. Klein *J. Chem. Phys*. **1994,** *101*, 4177-4189.

[16] W. G. Oover *Phys. Rev. A.* **1985**, *31*, 1695–1697.

[17] T. A. Darden, D. M. York, L. Pedersen, *J. Chem. Phys.* **1993**, *98*, 10089–10092.

[18] M. Tuckerman, G. J.Martyna, B. J. Berne *J Chem Phys.* **1992**, *97*, 1990-2001.
